# Depletion of ApoA5 aggravates spontaneous and diet-induced nonalcoholic fatty liver disease by reducing hepatic NR1D1 in hamsters

**DOI:** 10.7150/thno.91084

**Published:** 2024-02-24

**Authors:** Jiabao Guo, Guolin Miao, Wenxi Zhang, Haozhe Shi, Pingping Lai, Yitong Xu, Lianxin Zhang, Gonglie Chen, Yufei Han, Ying Zhao, Geroge Liu, Ling Zhang, Yuhui Wang, Wei Huang, Xunde Xian

**Affiliations:** 1 Institute of Cardiovascular Sciences, State Key Laboratory of Vascular Homeostasis and Remodeling, School of Basic Medical Sciences, Peking University, Beijing, China.; 2 Beijing Key Laboratory of Cardiovascular Receptors Research, Peking University Third Hospital, Beijing, China.; 3 Department of Urology, China-Japan Friendship Hospital, Beijing, China.

**Keywords:** Apolipoprotein A5, Syrian golden hamster, hypertriglyceridemia, nonalcoholic fatty liver disease, NR1D1

## Abstract

**Background:** ApoA5 mainly synthesized and secreted by liver is a key modulator of lipoprotein lipase (LPL) activity and triglyceride-rich lipoproteins (TRLs). Although the role of ApoA5 in extrahepatic triglyceride (TG) metabolism in circulation has been well documented, the relationship between ApoA5 and nonalcoholic fatty liver disease (NAFLD) remains incompletely understood and the underlying molecular mechanism still needs to be elucidated.

**Methods:** We used CRISPR/Cas9 gene editing to delete Apoa5 gene from Syrian golden hamster, a small rodent model replicating human metabolic features. Then, the ApoA5-deficient (ApoA5^-/-^) hamsters were used to investigate NAFLD with or without challenging a high fat diet (HFD).

**Results:** ApoA5^-/-^ hamsters exhibited hypertriglyceridemia (HTG) with markedly elevated TG levels at 2300 mg/dL and hepatic steatosis on a regular chow diet, accompanied with an increase in the expression levels of genes regulating lipolysis and small adipocytes in the adipose tissue. An HFD challenge predisposed ApoA5^-/-^ hamsters to severe HTG (sHTG) and nonalcoholic steatohepatitis (NASH). Mechanistic studies *in vitro* and *in vivo* revealed that targeting ApoA5 disrupted NR1D1 mRNA stability in the HepG2 cells and the liver to reduce both mRNA and protein levels of NR1D1, respectively. Overexpression of human NR1D1 by adeno-associated virus 8 (AAV8) in the livers of ApoA5^-/-^ hamsters significantly ameliorated fatty liver without affecting plasma lipid levels. Moreover, restoration of hepatic ApoA5 or activation of UCP1 in brown adipose tissue (BAT) by cold exposure or CL316243 administration could significantly correct sHTG and hepatic steatosis in ApoA5^-/-^ hamsters.

**Conclusions:** Our data demonstrate that HTG caused by ApoA5 deficiency in hamsters is sufficient to elicit hepatic steatosis and HFD aggravates NAFLD by reducing hepatic NR1D1 mRNA and protein levels, which provides a mechanistic link between ApoA5 and NAFLD and suggests the new insights into the potential therapeutic approaches for the treatment of HTG and the related disorders due to ApoA5 deficiency in the clinical trials in future.

## Introduction

A series of population-based studies have revealed that hypertriglyceridemia (HTG) with elevated plasma triglyceride (TG) levels is positive associated with the metabolic disorders, such as obesity, type II diabetes, nonalcoholic fatty liver disease (NAFLD) and acute pancreatitis 1-3, which recently has been recognized as an independent causal risk factor of cardiovascular disease (CVD) 4, 5. In China, the incidence of HTG is approximately 13.4%, which is much higher than that of hypercholesterolemia with 4.5% 6, seriously threatening human health and hindering the economic development.

At present, it is believed that the factors causing HTG can be divided into two aspects: environment and genetics, among which HTG caused by the former is mostly low and moderate, largely due to excessive intake of carbohydrates through dietary intervention 7, but the latter mainly through genetic mutations that impair lipoprotein lipase (LPL)-mediated hydrolysis of TG-rich lipoproteins (TRLs) 8, leading to a marked increase in circulating TG levels more than 1000 mg/dL and then forming severe HTG (sHTG). Unlike hypercholesterolemia that has been well defined in the past couple decades, the progress of the study on HTG has been relatively delayed because the *in vivo* metabolism of TG is complex, and how HTG, especially sHTG, contributes to metabolic disease still remains unclear.

Apolipoprotein A5 (ApoA5), a secretory protein with 39 kDa sorely synthesized by liver, is mainly carried on chylomicrons (CMs) and high-density lipoproteins (HDLs) 9. When compared to other apolipoproteins involved in TG metabolism, the abundance of ApoA5 is the lowest; however, humans and mice deficient with ApoA5 always exhibit HTG and moderate elevated TG levels 10, 11, respectively, suggesting that ApoA5 plays a central role in TG metabolism in circulation and could be a potential target to treat HTG and its related phenotypes. Unexpectedly, independent human studies with small sample size from different groups have shown that plasma ApoA5 levels were positively associated with TG concentration 12, 13. In addition, livers from patients with NAFLD, characterized by increased hepatic TG synthesis and accumulation at the early stage, had higher ApoA5 expression compared to controls 14. Ress et al reported that obese patients receiving bariatric surgery exhibit weight loss, improved insulin resistance and hepatic steatosis score with a reduction in ApoA5 mRNA 15. However, these observational studies cannot validate whether the changes in ApoA5 levels are the primary effects or the consequences of the compensatory actions in the diversely pathophysiological contexts. In agreement with the findings from human studies, the results using experimental mouse model showed that knockdown hepatic ApoA5 by an antisense oligonucleotide (ASO) led to reduced liver and muscle TG and diacylglycerol (DAG) to improve whole-body insulin resistance sensitivity under high fat diet condition, but unfortunately, ASO-treated mice only showed mildly elevated TG levels, which were not further aggravated by HFD 16, indicating that residual ApoA5 protein still executed its function. Furthermore, a study led by Sharma et al showed that AAV-mediated overexpression of human ApoA5 significantly reduce plasma TG levels, but the impact of ApoA5 overexpression on hepatic lipid contents in ApoA5^-/-^ mice has not been extensively investigated yet 17, making the relationship between HTG and NAFLD elusive.

Given that these previous findings observed in humans and mice demonstrated the paradoxical effects of ApoA5 on TG regulation and NAFLD, but mice partially or completely lacking ApoA5 only showed mildly or moderately elevated circulating TG concentration, which could not replicate HTG or sHTG in patients deficient with ApoA5, we applied CRISPR/Cas gene editing to target *Apoa5* gene from Syrian golden hamster, a small rodent with metabolic features similar to humans. We then identified the novel molecular mechanisms by which sHTG caused by ApoA5 deficiency in hamsters elicited NAFLD and explored the potential therapeutic approaches to treat sHTG-related disorders.

## Results

### Generation and characterization of ApoA5-deficient hamsters

**Figure 1:**
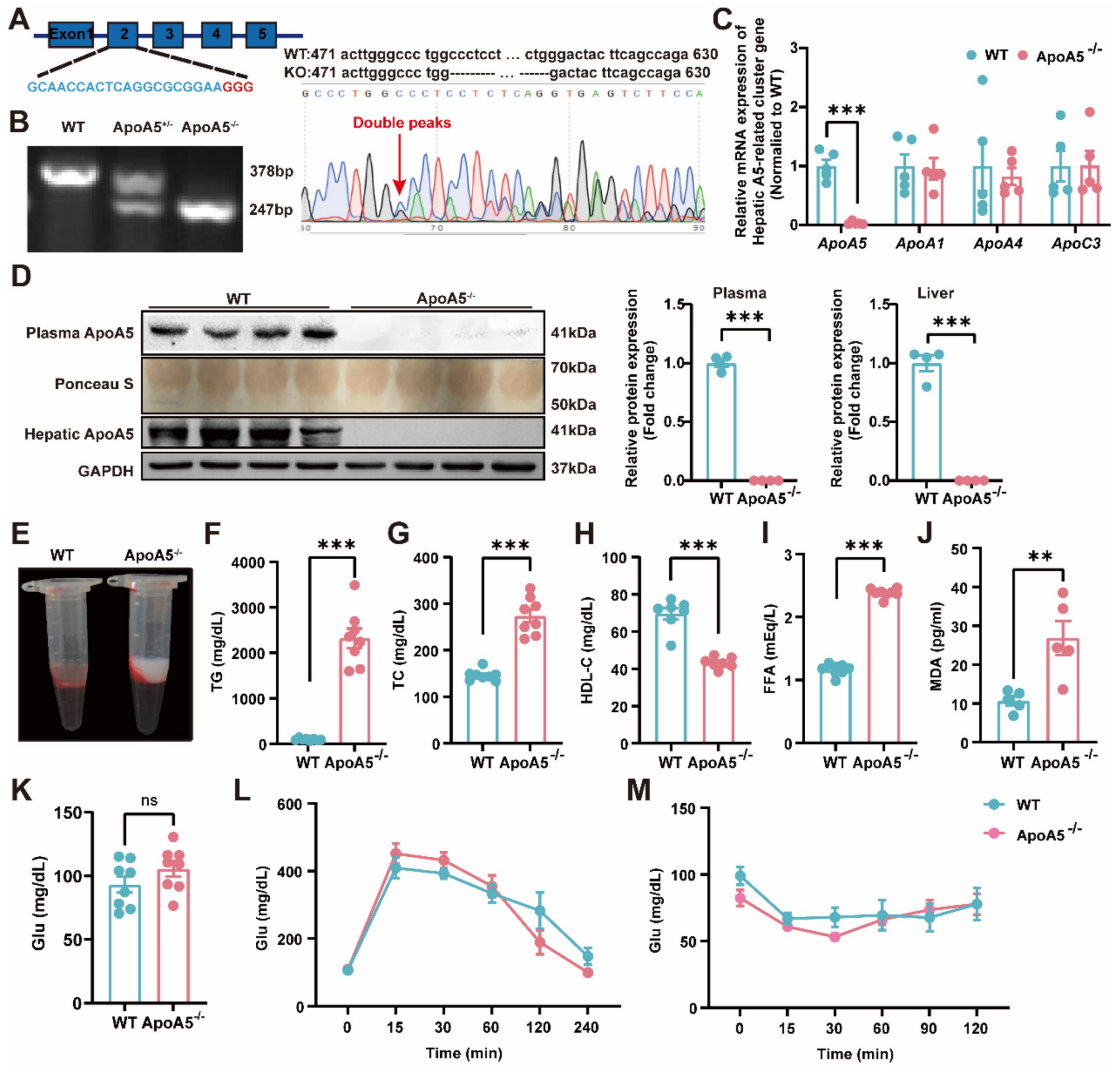
** Generation and characterization of ApoA5 knockout (ApoA5^-/-^) hamster model using CRISPR/Cas9 gene editing system.** A: Schematic of targeting the exon 2 of hamster *Apoa5* gene. The sgRNA and PAM sequences (GGG) are highlighted in blue and red, respectively. Sequencing of CRISPR/Cas9-edited *Apoa5* locus in Founder. Double peaks appeared at the mutation site. B: Genotyping results of F2 generations from wild type (WT), heterozygous (ApoA5^+/-^), and homozygous (ApoA5^-/-^) hamsters. C: mRNA expression of *Apoa5/a1/a4/c3* gene cluster in liver were determined by real-time PCR (n = 5/group). D: Representative Western blots of plasma and hepatic ApoA5 from 3-month-old male WT and ApoA5^-/-^ male hamsters on chow diet and quantitative data (n = 4/group). E: Representative images of plasma samples from 3-month-old male WT and ApoA5^-/-^ male hamsters on chow diet. F-K: Plasma triglyceride (TG) (F), total cholesterol (TC) (G), High density lipoprotein cholesterol (HDL-C) (H), free fatty acid (FFA) (I), malondialdehyde (MDA) (J) and glucose (Glu) (K) were determined from 3-month-old male WT and ApoA5^-/-^ hamsters on chow diet (n = 8/group). L-M: Glucose tolerance test (GTT) (L) and insulin tolerance test (ITT) (M) were performed on 3-month-old male WT and ApoA5^-/-^ hamsters on chow diet (n = 8/group). Error bars represent mean ± SEM. *P < 0.05; **P < 0.01; ***P < 0.001; ns, not significant.

To construct an ApoA5 deficient hamster model, we designed sgRNA targeting the exon 2 of hamster *Apoa5* gene. After microinjected with the designed sgRNA and Cas9 mRNA, zygotes were transplanted into the fallopian tube of surrogate hamsters. The sequencing data showed that one hamster pup displayed a 131 nt-deletion of the *Apoa5* gene (Figure 1A). The F2 animals were also genotyped to ensure the deletion of *Apoa5* gene was inheritable to offspring. As expected, wild-type (WT) and homozygous ApoA5-deficient (ApoA5^-/-^) hamsters showed 378 bp and 247 bp, respectively, while heterozygous knockout (ApoA5^+/-^) animals had both bands (Figure 1B). Moreover, we found that the mRNA level of *Apoa5* in liver was markedly reduced, while the mRNA levels of other apolipoproteins such as *Apoa1*, *Apoa4* and *Apoc3* on the same gene cluster were not altered (Figure 1C). Western blot revealed that plasma and liver ApoA5 levels were almost abolished in ApoA5^-/-^ hamsters compared to WT hamsters (Figure 1D). Since ApoA5 is an important agonist of plasma LPL to promote lipolysis 18. ApoA5^-/-^ hamsters on chow diet displayed milky plasma with markedly increased TG levels (Figure 1E, F), accompanied by elevated TC and FFA levels as well as reduced HDL-C level relative to the WT group (Figure 1G-I). In the meantime, a higher level of MDA, an indicator of lipid oxidation was observed in ApoA5^-/-^ hamsters (Figure 1J). However, the glucose level, glucose tolerance and insulin tolerance were identical between the two genotypes (Figure 1K-M), indicating that ApoA5 had no effect on glucose metabolism in our hamster model.

### ApoA5 deficiency causes abnormal synthesis, secretion and catabiosis of TG in hamsters

**Figure 2:**
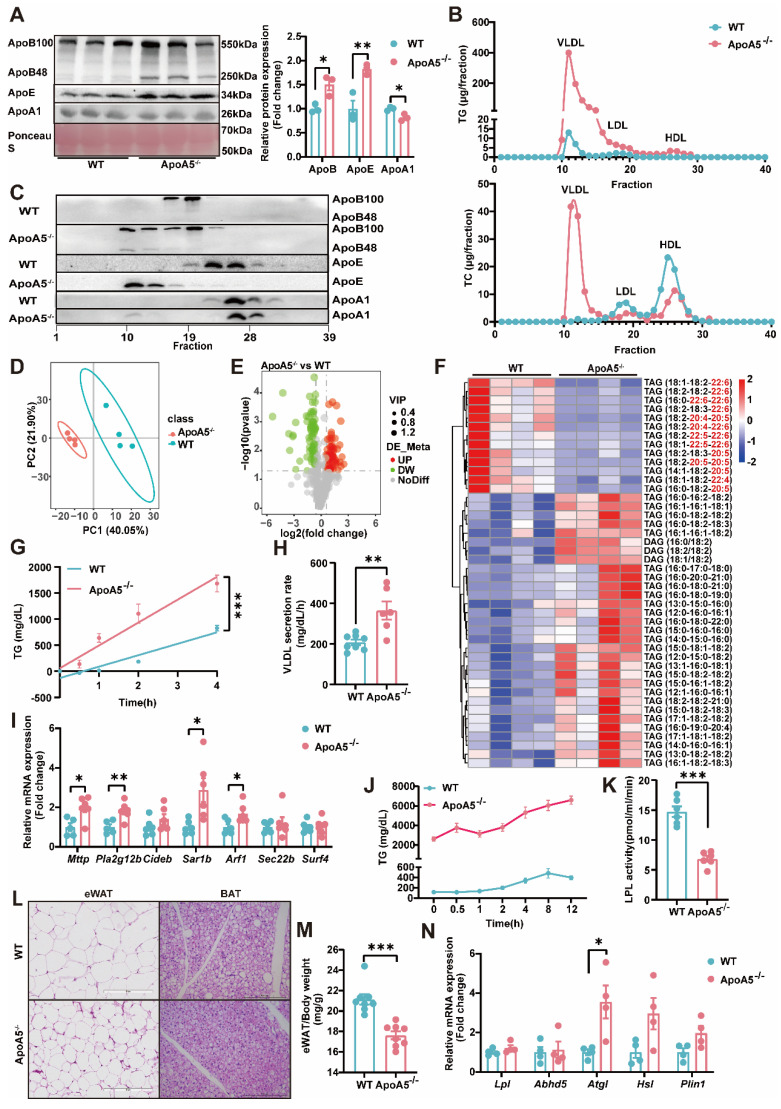
** The abnormal synthesis, secretion and catabiosis of triglycerides elicits severe hypertriglyceridemia in ApoA5^-/-^ hamsters under chow diet feeding.** A: Representative Western blots of plasma ApoB, ApoE and ApoA1 from 3-month-old male WT and ApoA5^-/-^ hamsters on chow diet and quantitative data (n = 3/group). B: Quantitative analysis of TG and TC contents in different fractions of pooled plasma analyzed by FPLC from 3-month-old male WT and ApoA5^-/-^ hamsters on chow diet. C: Representative Western blots of ApoB, ApoE and ApoA1 in each fraction described in (B) (n = 8/group). D-E: PCA analysis and volcano map of plasma lipidomics from the two groups described in (A) (n = 4/group). F: The heatmap of changed TAG/DAGs in the plasma lipidomics from the two groups described in (A) (n = 4/group). G-H: VLDL secretion was analyzed in hamsters after intraperitoneal injection of P-407 (1500 mg/kg) (n = 6-8/group). I: The expression levels of genes involved in VLDL secretion in liver were determined by real-time PCR (n = 6/group). J: Oral fat load test was conducted in the two genotypes described in (A). 12-hour fasted animals were gavaged with olive oil (10mL/kg) and plasma was collected at the indicated time points. TG concentrations were measured (n = 6/group). K: LPL activities were determined in post-heparin plasma in 3-month-old male WT and ApoA5^-/-^ hamsters on chow diet (n = 6/group). L: Representative HE stainings of epididymal white adipose tissue (eWAT) and brown adipose tissue (BAT) of 3-month-old male WT and ApoA5^-/-^ hamsters on chow diet. M: The ratio of eWAT weight and body weight from 3-month-old male WT and ApoA5^-/-^ hamsters on chow diet (n = 8/group). N: The expression levels of genes involved in lipolysis in eWAT were determined by real-time PCR (n = 4/group). Error bars represent mean ± SEM. *P < 0.05; **P < 0.01; ***P < 0.001; ns, not significant.

As TG metabolism is complex and the role of ApoA5 in the regulation of triglyceride metabolism is still not completely understood 19, we next detected the important apolipoproteins in plasma and found that the levels of ApoE and ApoB, representing VLDL and LDL, respectively, were significantly increased, while ApoA1 concentration was significantly decreased in ApoA5^-/-^ hamsters. Compared with WT hamsters, ApoA5^-/-^ hamsters showed that both ApoB100 and ApoB48 levels were increased markedly (Figure 2A). To further analyze the lipid distribution, we performed fast protein liquid chromatography (FPLC) and found that in ApoA5^-/-^ hamsters cholesterol content was increased significantly in VLDL fractions, which was decreased in LDL and HDL fractions, but TG content was increased over all fractions compared with WT group. Consistent with the results from plasma, more ApoB and ApoE localized on the VLDL fractions (Figure 2B and2C). Since HTG was the major phenotype in ApoA5^-/-^ hamsters, we performed lipidomics to investigate the changes in TG species (Figure 2D and 2E). TAGs containing polyunsaturated fatty acids (PUFAs) with more than 20 carbons, traditionally considered essential fatty acids obtained mainly from a routine diet, were decreased significantly, while TAG species containing saturated or monounsaturated fatty acids were increased in ApoA5^-/-^ hamsters on chow diet (Figure 2F). To explore the potential mechanisms by which ApoA5 deficiency caused elevated TG levels in hamsters, we injected P407, an LPL inhibitor, to examine hepatic VLDL secretion, and our data showed that the rate of VLDL secretion was significantly accelerated in ApoA5^-/-^ hamsters, while qPCR revealed a significant increase in the expression of *Mttp, Pla2g12b, Sar1b, Arf1*, the genes involved in VLDL secretion in the liver (Figure 2G-I). In addition, using the oral fat load assay, we found that ApoA5^-/-^ hamsters had a reduction in TG clearance, due to decreased LPL activity (Figure 2J and 2K). Furthermore, we also observed smaller white fat cells and brown fat cells, significantly reduced WAT mass, and increase in the mRNA expression level of *Atgl*, a key regulator of intracellular lipolysis, suggesting that ApoA5 deficiency enhanced TG catabolism, leading to reduced triglyceride accumulation in adipocyte tissues (Figure 2L-2N, Figure S1A and S1B). These results suggest that ApoA5 deficiency causes hypertriglyceridemia by impairing exogenous TG clearance in circulation, enhancing endogenous TG synthesis and secretion by liver, and reducing the storage capacity of adipose tissue.

### ApoA5 deficiency promotes hepatic steatosis in hamsters on chow diet

**Figure 3:**
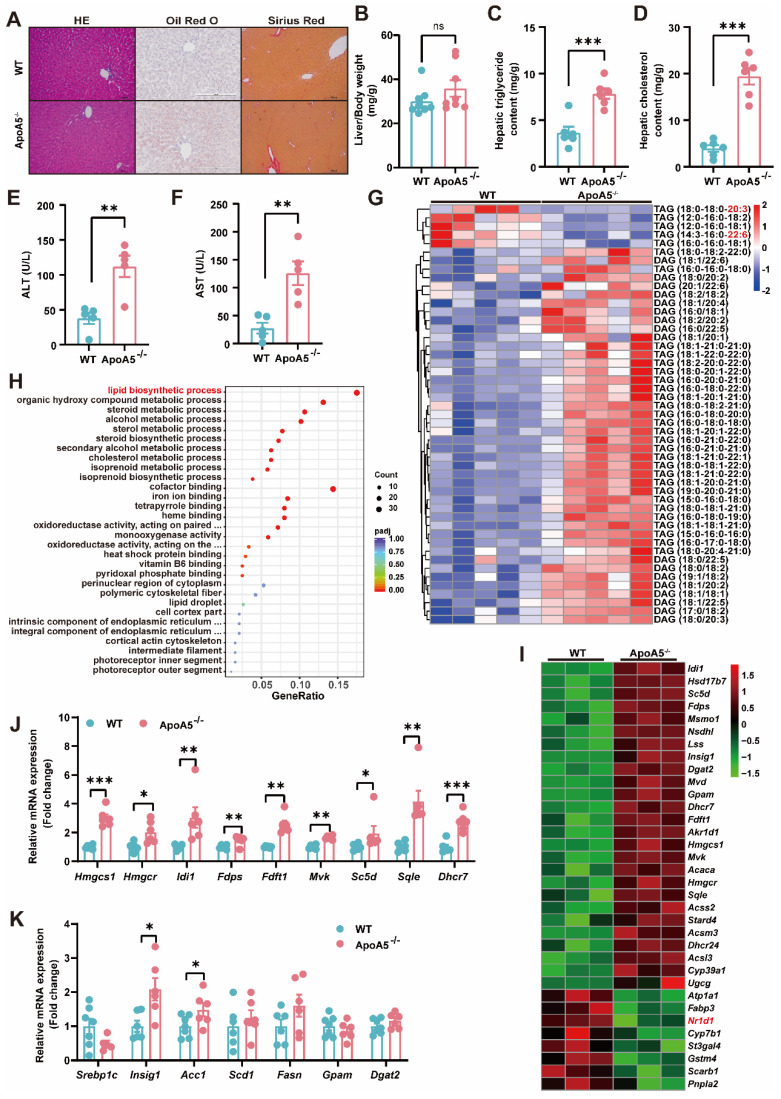
** ApoA5 deficiency causes hepatic lipid accumulation and hepatic steatosis in chow diet-fed hamsters.** A: The representative images of HE, Oil red O and Sirius red stainings in the liver sections of 3-month old male WT and ApoA5^-/-^ hamsters on chow diet. B: The ratio of liver weight and body weight from the animals described in (A) (n = 8/group). C-D: Hepatic lipid contents were measured and normalized to liver weight in the animals described in (A) (n = 6/group). E-F: ALT and AST determined from the animals described in (A) (n = 5/group). G: The heatmap of the changes in TAG/DAGs in the liver of the animals described in (A) (n = 5/group). H: GO pathway enrichment analysis of top 30 enriched pathways of Biological Process (BP), Molecular Function (MF) and Cellular Component (CC) of the transcriptome data in the livers of 3-month-old male WT and ApoA5^-/-^ hamsters on chow diet (n = 3/group). I: The heatmap of the changed genes involved in the lipid biosynthetic process from the transcriptome data in the liver of 3-month-old male WT and ApoA5^-/-^ hamsters on chow diet (n = 3/group). J: The expression levels of genes involved in cholesterol synthesis in the livers of 3-month-old male WT and ApoA5^-/-^ hamsters on chow diet were determined by real-time PCR (n = 6-7/group). K: The expression levels of genes involved in fatty acid synthesis in the livers of 3-month-old male WT and ApoA5^-/-^ hamsters on chow diet were determined by real-time PCR (n = 6-7/group). Error bars represent mean ± SEM. *P < 0.05; **P < 0.01; ***P < 0.001; ns, not significant.

Although elevated plasma triglyceride levels have been considered a risk factor of NAFLD, the relationship of ApoA5 and NAFLD is poorly understood. Under chow diet condition, we found that loss of ApoA5 had no significant impact on the morphology of liver cells and liver weight (Figure 3A and 3B). However, ApoA5^-/-^ hamsters showed markedly increased lipid accumulation in the livers and elevated plasma ALT and AST levels, indicating liver dysfunction (Figure 3A, 3C-3F); however, no change in fibrosis analyzed by sirius red staining showed in ApoA5^-/-^ hamsters when compared with WT animals (Figure 3A). Further analysis using lipidomics showed an increase in TAGs containing saturated or monounsaturated fatty acids in the livers of ApoA5^-/-^ hamsters (Figure 3G). In agreement with the pathological and lipidomics findings, the transcriptomics data also revealed an increase in the hepatic lipid biosynthesis process in the absence of ApoA5, in which the expression of genes regulating lipid synthesis, especially cholesterol synthesis, was increased, whereas the expression of nuclear transcription factor *Nr1d1* was decreased, which was consistent with previous results that NR1D1 regulated cholesterol synthesis pathway (Figure 3H and 3I) 20. In addition to cholesterol synthesis, the expression of genes responsible for the fatty acid synthesis pathway was also increased (Figure 3J and 3K). Our data demonstrate that ApoA5 deficiency promotes increased lipid accumulation by increasing hepatic lipid synthesis, then leading to hepatic steatosis in hamsters on chow diet.

### ApoA5 inactivation exacerbates high fat diet (HFD)-induced NASH in hamsters

**Figure 4:**
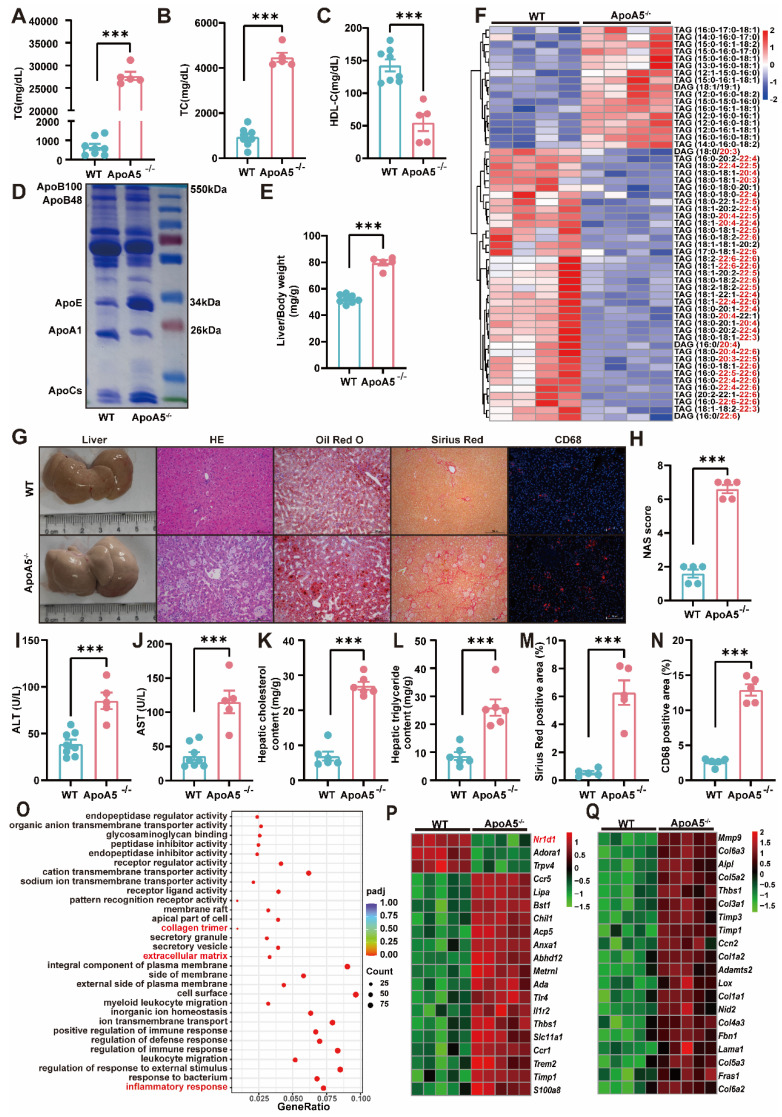
** High fat diet (HFD)-induced hepatic steatosis, inflammation and fibrosis are exacerbated in ApoA5^-/-^ hamsters.** A-C: Plasma TG (A), TC (B) and HDL-C (C) were determined from HFD-fed WT and ApoA5^-/-^ hamsters for 12 weeks (n = 5-8/group). D: The representative SDS-PAGE gel of plasma lipid depleted lipoproteins (d < 1.063) from WT and ApoA5^-/-^ hamsters on HFD. E: The ratio of liver weight and body weight from the animals described in (A-C) (n = 5-8/group). F: The heatmap of the changes TAG/DAGs in the livers of the animals described in (A-C) (n = 4/group). G: The representative images of livers and the stainings of HE, Oil red O, Sirius red, and CD68 in liver sections of WT and ApoA5^-/-^ hamsters on HFD. H: Quantification of NAFLD activity score (NAS) of the livers of WT and ApoA5^-/-^ hamsters on HFD (n = 5/group). I-J: Determination of ALT and AST from WT and ApoA5^-/-^ hamsters on HFD (n = 5~8/group). K-L: Analysis of hepatic lipids contents and the ratio of liver/body weight of WT and ApoA5^-/-^ hamsters on HFD (n = 6/group). M: Quantification of Sirius red-positive areas in the livers of WT and ApoA5^-/-^ hamsters on HFD (n = 5/group). N: Quantification of CD68-positive areas in the livers of WT and ApoA5^-/-^ hamsters on HFD (n = 5/group). O: GO pathway enrichment analysis of top 30 enriched pathways of BP, MF and CC of the transcriptome data in the livers of WT and ApoA5^-/-^ hamsters on HFD (n = 5/group). P-Q: The heatmap of the changed genes involved in the inflammatory response and fibrosis from the transcriptome data in the livers of WT and ApoA5^-/-^ hamsters on HFD (n = 5/group). Error bars represent mean ± SEM. *P < 0.05; **P < 0.01; ***P < 0.001; ns, not significant.

To our knowledge, WT hamster is susceptible to the high fat diet treatment, and diet-induced NAFLD 21. It was rational for us to investigate whether HFD could further worsen the degree of fatty liver. After fed an HFD containing 20% fat, 0.5% cholesterol for 3 months, ApoA5^-/-^ hamsters showed severe combined hyperlipidemia with triglyceride levels more than 25,000 mg/dL and total cholesterol levels higher than 4000 mg/dL, while HDL-C levels were still lower in ApoA5^-/-^ hamsters than WT hamsters (Figure 4A-4C). To avoid FPLC column blockage caused by very large lipoprotein particles, we purified total plasma lipoprotein and subjected to SDS-PAGE gel, which showed that ApoB and ApoE levels were significantly increased, but ApoA1 level was reduced in ApoA5^-/-^ hamsters compared with WT hamsters (Figure 4D). Moreover, the phenotype of hepatomegaly was only observed in HFD-fed ApoA5^-/-^ hamsters (Figure 4E and 4G), which was similar to the manifestations reported in some ApoA5-deficient patients 22. Liver lipidomics data consistently confirmed that TAG species containing PUFAs were reduced, and TAGs containing saturated or monounsaturated fatty acids were significantly increased in ApoA5^-/-^ hamsters fed HFD for 3 months (Figure 4F).

HE staining in the livers illustrated that ApoA5^-/^ hamsters had a significant increase in inflammatory aggregates, the number of lipid droplets, and NAFLD activity score (NAS), and as well as higher plasma ALT and AST levels (Figure 4G-4J). Additionally, oil red O staining and the quantitative data demonstrated that hepatic lipids, including TG and TC, were largely deposited in the livers (Figure 4G, 4K and 4L), together with obvious liver fibrosis of ApoA5^-/-^ hamsters (Figure 4M). CD68 immunofluorescence staining showed increased macrophage infiltration, indicating aggravated inflammation in the liver (Figure 4G and 4N). Similarly, our results from liver transcriptomics and qPCR suggested that ApoA5 deficiency coordinating with HFD promoted an activation of the inflammatory and fibrotic pathways, then leading to NASH (Figure 4O-4Q; Figure S2A and 2B). HTG and NASH have been reported to be positively associated with the incidence of atherosclerosis 23; however, surprisingly, inactivation of ApoA5 had no effect on spontaneous atherosclerosis in both 8-month old and 18-month old hamsters on chow diet, but only mild atherosclerotic lesions were observed in HFD fed animals (Figure S3), indicating that NAFLD, rather than atherosclerotic cardiovascular disease (ASCVD), was the major event in the setting of ApoA5 deficiency in our hamster model.

### Depletion of ApoA5 aggravates NASH by reducing NR1D1 expression

**Figure 5:**
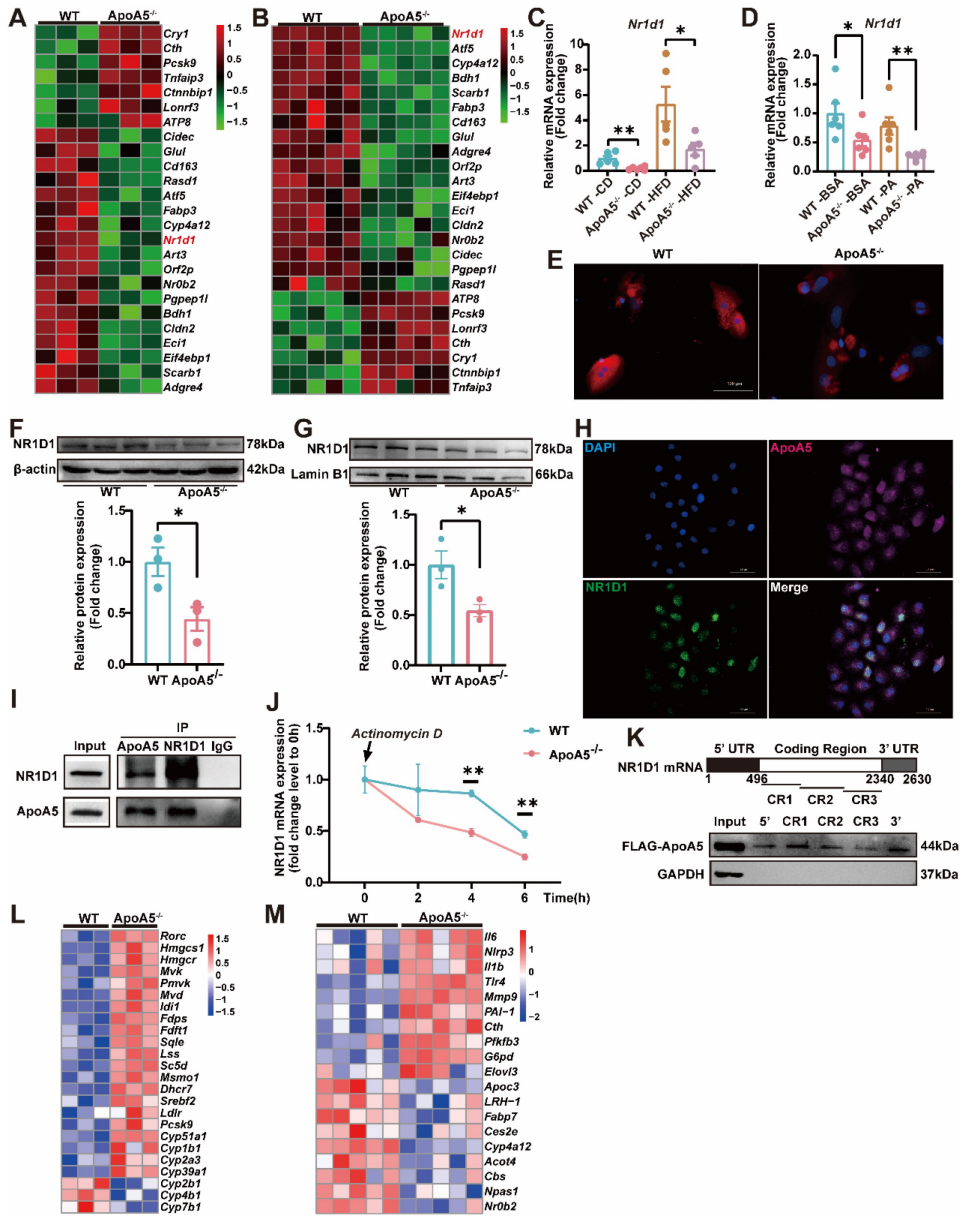
** NR1D1 expression and mRNA stability are decreased in ApoA5^-/-^ hamsters under different nutrient conditions.** A-B: The heatmap analysis of the genes, which mRNA expression levels were altered in the livers of both CD (A) and HFD-fed (B) WT and ApoA5^-/-^ hamsters. C: The mRNA levels of NR1D1 in the livers of WT and ApoA5^-/-^ hamsters under CD and HFD (n = 5-6/group). D: The mRNA levels of NR1D1 in the cultured primary hepatocytes of WT and ApoA5^-/-^ hamsters treated with BSA or PA (500 μM) (n = 6/group). E: The representative immunofluorescence images of NR1D1 in the cultured primary hepatocytes of WT and ApoA5^-/-^ hamsters. F: Western blot analysis of NR1D1 protein in the liver samples of CD-fed WT and ApoA5^-/-^ hamsters and quantitative data (n = 3/group). G: Western blot analysis of NR1D1 in the nucleus of liver samples of CD-fed WT and ApoA5^-/-^ hamsters and quantitative data (n = 3/group). H: The representative immunofluorescence images of NR1D1 and ApoA5 in HepG2 cells. I: Co-IP analysis of the interaction between ApoA5 and NR1D1 in the livers of CD-fed hamster. J: Analysis of NR1D1 mRNA levels in the cultured primary hepatocytes from the two genotypes after treated with Actinomycin D (2 μg/mL) (n = 3/group). K: RNA pulldown assays were performed by using HepG2 cell lysates and in vitro-transcribed RNAs depicted to test the binding of ApoA5 to *Nr1d1* mRNA. HepG2 cells were transfected with ApoA5 plasmid containing a FLAG tag for 48 h. L-M: The heatmap of the changed downstream genes of NR1D1 in the transcriptome data in the livers of WT and ApoA5^-/-^ hamsters under CD (C) and HFD (D) conditions. Error bars represent mean ± SEM. *P < 0.05; **P < 0.01; ***P < 0.001; ns, not significant.

To explore the potential molecular mechanism by which ApoA5 deficiency leads to fatty liver disease, we further analyzed liver transcriptomics data and identified 25 genes with the same trend in the liver of hamsters fed chow diet and HFD (Figure 5A, B). Among them, NR1D1, a nuclear transcription factor that regulates lipid metabolism, inflammation, and fibrosis 24, 25, was significantly reduced in ApoA5^-/-^ hamsters, and the reduction was the most significant in the transcriptomics under HFD feeding condition. We then examined *Nr1d1* gene expression levels in hamster liver and primary hepatocytes, which were consistently reduced under both normal and high fat conditions (Figure 5C and 5D). *In vitro* immunofluorescence assay of NR1D1 in primary hepatocytes and Western blot analysis of hepatic NR1D1 also showed a reduction in NR1D1 protein levels in the absence of ApoA5 (Figure 5E and 5F, Figure S4A). Similarly, knockdown of ApoA5 expression by siRNA in HepG2 cells reduced the mRNA and protein levels of NR1D1 (Figure S4B-S4E). In addition, as a transcription factor that functions mainly in the nucleus, we found that NR1D1 protein levels were significantly reduced in nucleus of the primary hepatocytes and HepG2 cells lacking ApoA5 (Figure 5G and Figure S4F-S4G). Meanwhile, we found that ApoA5 could be detected in nucleus of HepG2 cells (Figure S4F). To validate the localization in the compartment of HepG2 cells, we performed immunofluorescence assay and our data showed that although ApoA5 and NR1D1 were mainly present in the cytoplasm and nucleus, respectively, these two proteins also partially co-localized in nucleus (Figure 5H). To clarify the relationship between ApoA5 and NR1D1, we conducted Co-IP experiments using liver samples and observed an interaction between ApoA5 and NR1D1(Figure 5I) and we proposed that the ApoA5/NR1D1 complex might translocate to the nucleus to exert its function. To examine the homeostatic regulation of NR1D1 proteins, we first treated HepG2 cells with cycloheximide (CHX) to analyze protein stability. We observed substantial degradation of NR1D1 over a 12-hour period, which was largely blocked by proteasome inhibitor MG132. However, the knockdown of ApoA5 had no effect on the degradation velocity of NR1D1 (Figure S4H-S4J), suggesting that ApoA5 did not regulate the protein stability of NR1D1. Furthermore, we found that the mRNA stability of NR1D1 was significantly reduced in ApoA5-deficient hepatocytes, possibly contributing to the reduced NR1D1 mRNA levels and then NR1D1 protein levels (Figure 5J). Similarly, knockdown of ApoA5 by siRNA in HepG2 cells impaired mRNA stability of NR1D1 and overexpression of ApoA5 by plasmid in HepG2 cells improved mRNA stability of NR1D1(Figure S4L-S4M). To further investigate the mechanisms underlying ApoA5-mediated *Nr1d1* mRNA stability, we performed RNA pulldown assays. As shown in Figure 5K, ApoA5 was found to bind to the *Nr1d1* mRNA at multiple sites, implying that ApoA5 could regulate NR1D1 expression through a direct binding to *Nr1d1* mRNA. Furthermore, we also performed ChIP assays to examine that whether ApoA5 could regulate the transcription of *Nr1d1*, the result showed that ApoA5 had no interaction with *Nr1d1* promoter (Figure S4N), indicating that ApoA5 deficiency did not impair the transcriptional function of *Nr1d1*. To confirm the functional role of ApoA5 in NR1D1, we analyzed the transcriptomics of the livers from WT and ApoA5^-/-^ hamsters, which showed that the mRNA expression of NR1D1-regulated genes participating in lipid metabolism, inflammation, and fibrosis was significantly upregulated in ApoA5^-/-^ hamsters upon different dietary interventions (Figure 5L and 5M), suggesting that ApoA5 may be a crucial indicator of the progression of NAFLD through regulating the proper function of NR1D1.

### AAV8-mediated overexpression of NR1D1 ameliorates hepatic steatosis caused by ApoA5 deficiency

**Figure 6:**
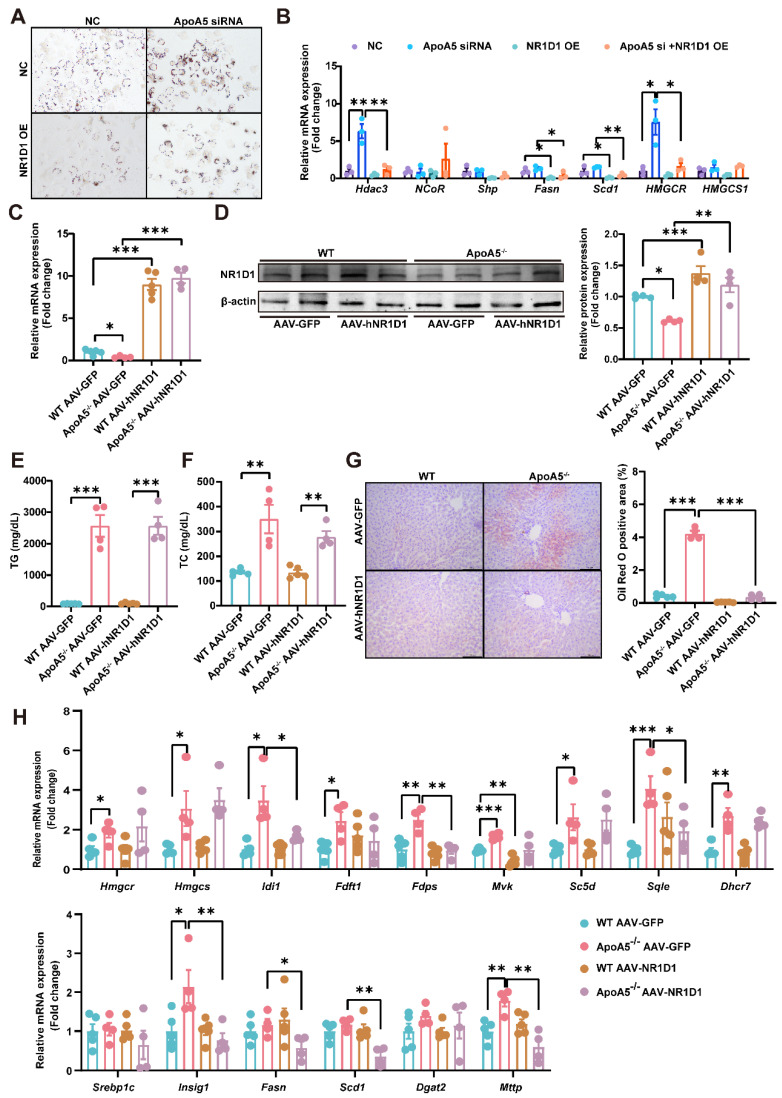
** Overexpression of human NR1D1 reverses hepatic lipid accumulation induced by ApoA5^-/-^ deficiency *in vitro* and *in vivo*.** A: Representative images of Oil red O staining in HepG2 cells after ApoA5 knockdown and/or NR1D1 overexpression. B: The expression levels of NR1D1 downstream genes involved in lipid synthesis in HepG2 cells after ApoA5 knockdown and/or NR1D1 overexpression were determined by real-time PCR (n = 3/group). C: The mRNA levels of NR1D1 in the livers of CD-fed WT and ApoA5^-/-^ hamsters overexpressing NR1D1 by AAV8 for 4 weeks (n = 4-5/group). D: The protein level of NR1D1 in the liver of WT and ApoA5^-/-^ hamsters overexpressing NR1D1 by AAV8 (n = 4/group). E-F: Plasma TG (E) and TC (F) determined from WT and ApoA5^-/-^ hamsters overexpressing NR1D1 by AAV8 (n = 4-5/group). G: The representative images of Oil red O staining in liver sections of WT and ApoA5^-/-^ hamsters overexpressing NR1D1 by AAV8 and quantitative data (n = 4-5/group). H: The expression levels of genes involved in cholesterol and fatty acid synthesis in the livers of the animals described in (C) were determined by real-time PCR (n = 4-5/group). Error bars represent mean ± SEM. *P < 0.05; **P < 0.01; ***P < 0.001; ns, not significant.

To further clarify the role of NR1D1 in hepatic steatosis caused by ApoA5 deficiency, we overexpressed NR1D1 in HepG2 cells after knocking down ApoA5 and treated with 500μM palmitic acid for 16 h. Oil red O staining showed that overexpression of human NR1D1 (hNR1D1) ameliorated the lipid accumulation caused by ApoA5 deficiency (Figure 6A). The mRNA expression of *Hdac3* in the NR1D1 functional complex was up-regulated after knockdown of ApoA5, but down-regulated after NR1D1 overexpression 26. Consistently, the expression levels of genes involved in fatty acid and cholesterol synthesis were also significantly decreased after overexpressing hNR1D1 (Figure 6B). We then validated the beneficial effect of hNR1D1 *in vivo* by AAV8 injection and found that although there was no significant reduction in blood lipids (Figure 6C-6F), lipid accumulation in the liver was improved after hNR1D1 overexpression for 4 weeks in ApoA5^-/-^ hamsters on chow diet (Figure 6G), and the mRNA expression of genes related to cholesterol synthesis and fatty acid synthesis was down-regulated under hNR1D1 overexpression condition (Figure 6H). The above results indicate that increased NR1D1 levels is sufficient to protect against hepatic steatosis caused by ApoA5 depletion independent of plasma lipid profiling.

### Restoration of human ApoA5 by AAV8 rescues hypertriglyceridemia and hepatic steatosis in ApoA5^-/-^ hamsters on chow diet

**Figure 7:**
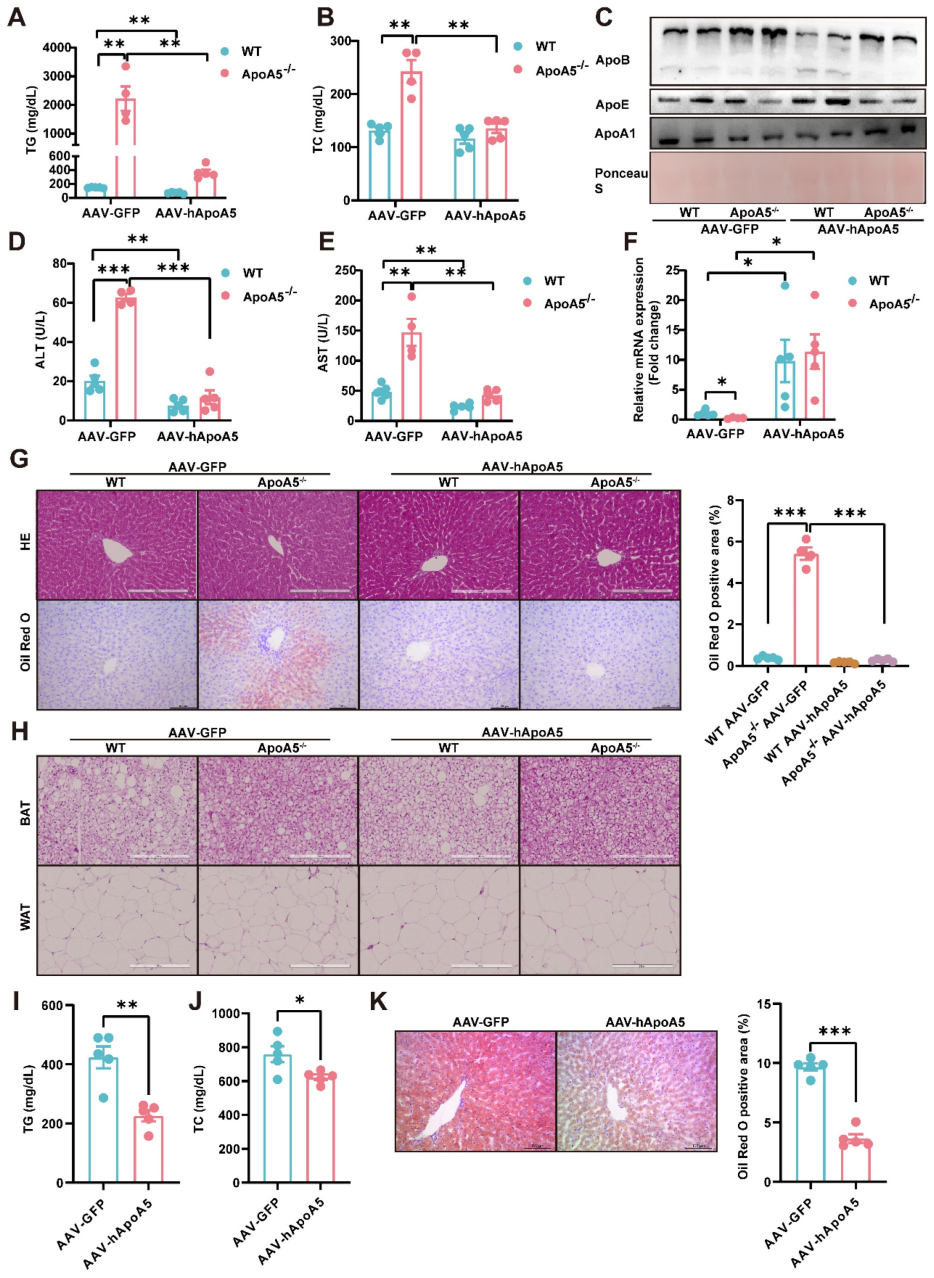
** Re-expression of human ApoA5 (hApoA5) by AAV rescues hyperlipidemia and hepatic steatosis in ApoA5-deficient hamsters on chow diet.** A-B: Plasma TG (A) and TC (B) were determined from CD-fed WT and ApoA5^-/-^ hamsters re-expressing hApoA5 by AAV (n = 4-5/group). C: Representative Western blots of plasma ApoB, ApoE and ApoA1 from the animals described in (A-B). D-E: Determination of ALT and AST from the animals described in (A-B) (n = 4-5/group). F: The mRNA levels of NR1D1 in the livers of the animals described in (A-B) (n = 4-5/group). G: The representative images of HE and Oil red O stainings in liver sections of the animals described in (A-B) and quantitative data (n = 4-5/group). H: The representative images of HE staining in eWAT and BAT sections of the animals described in (A-B). I-J: Plasma TG (I) and TC (J) were determined from WT hamsters feeding HFD for 4 weeks and overexpressing hApoA5 by AAV8 for 2 weeks (n = 5/group). K: The representative images of Oil red O stainings in liver sections of the animals described in (I-J) and quantitative data (n = 5/group). Error bars represent mean ± SEM. *P < 0.05; **P < 0.01; ***P < 0.001; ns, not significant.

Given that the overexpression of hNR1D1 had no beneficial effect on HTG in ApoA5^-/-^ hamsters, which still may predispose animals to the metabolic disease, making us consider to restore ApoA5 to rescue hypertriglyceridemia and fatty liver due to ApoA5 deficiency. Administration of AAV8-human ApoA5 (hApoA5) completely corrected plasma TG and TC in ApoA5^-/-^ hamsters (Figure 7A and 7B). The levels of plasma ApoB and ApoE were also decreased, accompanied by increased ApoA1 levels when compared to AAV8-GFP treated ApoA5^-/-^ hamsters (Figure 7C). In the meantime, plasma ALT and AST levels were reduced, indicating an improvement of liver function in ApoA5^-/-^ hamsters after ApoA5 restoration *in vivo* (Figure 7D and 7E). Additionally, the mRNA level of NR1D1 in the liver was also increased in ApoA5^-/-^ hamsters receiving AAV8-mediated ApoA5 re-expression, further supporting the concept that ApoA5 was a new regulator of NR1D1 (Figure 7F). As expected, HE staining showed no significant changes in liver morphology, but oil red O staining demonstrated that hepatic lipid accumulation was significantly reduced after ApoA5 restoration (Figure 7G). In addition, compared with AAV8-GFP treated ApoA5^-/-^ hamsters, AAV8-hApoA5 administrated ApoA5^-/-^ animals showed larger white adipocytes with less crown like structure (CLS) in WAT, suggesting that the lipid storage capacity of adipose tissue was restored and reduced inflammatory response after ApoA5 supplementation (Figure 7H). To further confirm the therapeutic significance of ApoA5, we applied AAV8 to overexpress hApoA5 in the livers of HFD-fed WT hamsters for 2 weeks and found that hepatic ApoA5 overexpression completely corrected plasma TG elevation and partly reduced plasma TC (Figure 7I and 7J). Oil red O staining showed that hepatic lipid accumulation was significantly reduced after hApoA5 overexpression (Figure 7K). Collectively, these results suggested that restoration of ApoA5 in knockout hamsters not only ameliorated ApoA5 deficiency-induced hypertriglyceridemia and hepatic steatosis, but also rescued the abnormalities in other metabolic tissues.

### Activation of BAT and WAT improves hyperlipidemia in ApoA5^-/-^ hamsters under different dietary interventions

**Figure 8:**
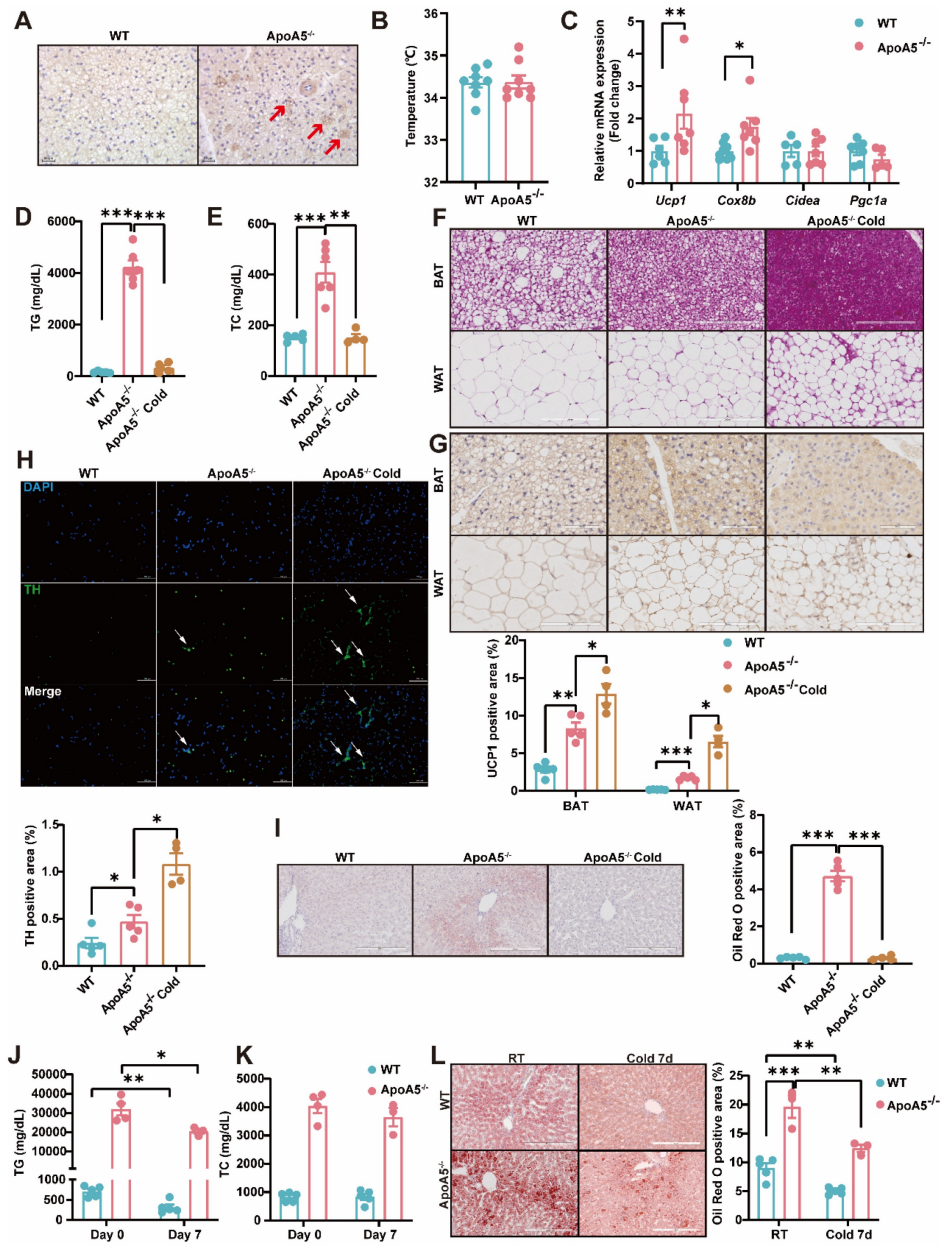
** Cold exposure improves lipid metabolism disorders caused by ApoA5 deficiency.** A: The representative images of UCP1 immunohistochemical staining in BAT sections of 3-month old male WT and ApoA5^-/-^ hamsters on chow diet. B: The body temperature of the hamsters described in (A) (n = 8/group). C: The expression levels of genes involved in thermogenesis in the BAT of the hamsters described in (A) (n = 5-7/group). D-E: Plasma TG (D) and TC (E) were determined from WT and ApoA5^-/-^ hamsters after cold exposure for 5 days (n = 4-6/group). F-G: The representative images of HE and UCP1 immunohistochemical staining in BAT and eWAT sections of the animals described in (D-E) and quantitative data (n = 4-6/group). H: The representative images of tyrosine hydroxylase (TH) immunofluorescence staining in eWAT sections of the hamsters described in (D-E) and quantitative data (n = 4-6/group). I: The representative images of Oil red O in liver sections of the hamsters described in (D-E) and quantitative data (n = 4-6/group). J-K: Plasma TG (J) and TC (K) were determined from WT and ApoA5^-/-^ hamsters on HFD after cold exposure for 7 days (n = 3-5/group). L: The representative images of Oil red O in liver sections of the hamsters described in (J-K) and quantitative data (n = 3-5/group). Error bars represent mean ± SEM. *P < 0.05; **P < 0.01; ***P < 0.001; ns, not significant.

Based on our observations described above that ApoA5 plays a crucial role in lipid metabolism in adipose tissue, we proposed an organ crosstalk between liver and adipose tissue. Immunohistochemical staining showed that the expression of UCP1 in brown fat was increased in ApoA5^-/-^ hamsters on chow diet (Figure 8A), and the transcriptional expression of the thermogenic genes was also up-regulated (Figure 8C). However, the rectal temperature of WT and ApoA5^-/-^ hamsters was similar (Figure 8B), indicating that ApoA5^-/-^ hamsters need more thermogenesis to maintain body temperature through an unknown compensatory pathway, which may be attributed to the impaired function of adipose tissue. According to the previous studies, cold exposure can improve lipid metabolism in animals 27. When exposed to cold treatment at 4 °C for 5 days, ApoA5^-/-^ hamsters showed that the plasma TG and TC levels were completely normalized as the levels of WT hamsters (Figure 8D and 8E). In consistent, the plasma ApoB and ApoE levels were also markedly decreased, while the plasma ApoA1 level was increased (Figure S5A). FPLC analysis further confirmed that the lipid profile of ApoA5^-/-^ hamsters after cold exposure was similar to that of WT hamsters (Figure S5B). Moreover, the weight of WAT and BAT was significantly decreased, while the weight of liver was slightly increased after cold exposure compared to WT hamster and ApoA5^-/-^ hamsters without cold treatment (Figure S5C). After cold exposure, adipose tissue of ApoA5^-/-^ hamsters showed that BAT was denser and more metabolically active, while WAT switched to a beige phenotype (Figure 8F and 8G). Immunofluorescence staining of tyrosine hydroxylase (TH) in WAT, a marker of sympathetic nerve, showed that the basal TH level was increased immediately in the absence of ApoA5, which was further increased after cold exposure (Figure 8H), suggesting that the cold stimulation may regulate lipid metabolism and thermogenesis through sympathetic nerves. In addition, the lipid accumulation was significantly reduced in the liver upon cold exposure (Figure 8I). Next, to further investigate the therapeutic effect of cold exposure on lipid metabolism in ApoA5^-/-^ hamsters under HFD feeding condition, hamsters fed with an HFD for 4 weeks were exposed to intermittent 6 h/d cold exposure for 7 days. Severe HTG and hepatic lipid accumulation were partially rescued in both WT and ApoA5^-/-^ hamsters (Figure 8J-8L). Meanwhile, we also used the β3-AR agonist CL316243 to activate BAT for 4 weeks and found that subcutaneous administration of CL316243 resulted in the changes in plasma lipids in ApoA5^-/-^ hamsters similar to the findings observed in the cold treated animals (Figure S6A-6D). In addition, the morphological changes of adipocytes and thermogenesis in BAT and WAT are similar to the outcomes of cold exposure (Figure S6E and 6G). So far, IL-6, a secretory cytokine mainly executing pro-inflammatory function, has been reported to regulate thermogenesis 28. To verify whether IL-6 level was altered in the setting of ApoA5 deficiency, we performed immunofluorescence staining showing increased IL6 level in BAT of ApoA5^-/-^ hamsters, which may account for the increased thermogenesis in ApoA5^-/-^ hamsters (Figure S6H). Moreover, CD68 staining suggested that adipose tissue from ApoA5^-/-^ hamsters was in a low-grade inflammation environment with elevated IL-6 concentration, which was aggravated by adipose activation (Figure S6F). Liver oil red O staining showed that activation of adipose tissue by CL316243 also improved liver lipid accumulation (Figure S6I). These results suggest that activation of adipose tissue can improve lipid metabolism disorders and hepatic steatosis caused by ApoA5 deficiency.

## Discussion

The relationship between ApoA5 and plasma TG metabolism has been well established; however, the influence of ApoA5 on NAFLD and atherosclerotic cardiovascular disease (ASCVD) has remained largely elusive. In the present study, we created a new Syrian golden hamster model lacking *Apoa5* gene using CRISPR/Cas9 gene editing to demonstrate that loss of endogenous ApoA5 caused HTG and hepatic steatosis under chow diet condition and HFD aggravated the development of NAFLD through regulating circadian gene *Nr1d1*, providing a new molecular mechanism by which ApoA5 protested against NAFLD; however, these metabolic phenotypes has no or minor effects on atherogenesis on different dietary interventions, respectively. We also revealed that overexpression of human NR1D1 or re-expression of human ApoA5 by AAV8 effectively improved lipid accumulation in the livers of ApoA5^-/-^ hamsters independent on plasma lipid profiling. Moreover, ApoA5 depletion elicited low grade chronic inflammation with increased cytokine IL-6 to enhance lipolysis in WAT and activate thermogenesis in BAT, and activation of BAT by cold exposure or CL316243 significantly ameliorated HTG and NAFLD. Therefore, these data clarify a beneficial role of ApoA5 in NAFLD.

Although murine model lacking ApoA5 has been generated, the phenotype and mechanism of NAFLD are still poorly understood partially because plasma triglyceride levels are only elevated by 2-4 folds and mice are resistant to HFD-induced HTG 10. Previously, using ASO to knock down hepatic ApoA5 in mice, Camporez et al reported that ASO-treated mice showed a moderate increase in plasma TG levels, which could not be further increased by an HFD challenge. Unexpectedly, mice with less ApoA5 levels had reduced hepatic TG content on both chow diet and HFD feeding conditions, but displayed improved HFD-induced insulin resistance, which was probably attributable to reduced DAG concentration in the liver 16.

Moreover, recent study by Li and the colleagues demonstrated that olanzapine, a drug used for treating schizophrenia, increased intracellular ApoA5 levels by enhancing the interaction of ApoA5 and sortilin to impair its transport, then leading to hepatic steatosis 29; however, olanzapine treatment significantly increased plasma TG and TC concentration, accompanied with elevated AST and ALT levels. These observations from the independent studies suggest that ApoA5 plays a detrimental role in liver and silencing hepatic ApoA5 could be a potential therapeutic approach of fatty liver. In contrast, our model herein showed that targeting ApoA5 led to severe HTG similar to ApoA5-deficient patients, overt hepatic lipid accumulation and liver injury in hamsters fed a chow diet without affecting glucose metabolism. ApoA5^-/-^ hamsters also had impaired TG-rich lipoprotein clearance from the circulation and enhanced VLDL secretion, both contributing to the apparent hypertriglyceridemic phenotype. Consistently, lipidomics study revealed that the levels of TAG species containing saturated or monounsaturated fatty acids, representing endogenous lipids, were higher, whereas the concentration of TAGs with PUFAs, representing exogenous lipids from diet, was significantly decreased in ApoA5^-/-^ hamsters, suggesting that ApoA5 is an essential modulator of extracellular and intracellular lipid metabolism. The discrepancy of hepatic steatosis between our and other studies could be explained by the several possibilities: 1) mice treated with ASO still had 30-40% of endogenous ApoA5 protein, while ApoA5 protein in knockout hamsters was completely abolished, indicating that the residual ApoA5 prevented lipid accumulation through an uncovered compensatory mechanism; 2) The plasma lipid levels of ApoA5-deficient mice and hamsters without any dietary intervention were not comparable because the former only showed mild to modest elevation of TG, but the latter had severe HTG with increased TC and decreased HDL-C; however, HFD aggravated severe HTG and liver injury only in hamsters, but not in mice; 3) although olanzapine inhibited ApoA5 secretion, leading to increased hepatic ApoA5 level, the link between ApoA5 and hepatic steatosis was still missing because the effect of olanzapine on fatty liver in ApoA5-deficnent mice had not been investigated yet, making the relationship between ApoA5 and NAFLD unclear.

Population-based on studies has demonstrated that HTG is positively associated with fatty liver in the context of obesity and lipodystrophy 30. In the present study, we reported for the first time that ApoA5 deficiency caused small adipocytes in WAT and BAT with less lipid droplets and more crown-like structure (CLS), indicating that lipolysis was enhanced and the lipid storage capacity was reduced in adipose tissue. Moreover, we also observed a remarkable increase in the level of thermogenesis-related uncoupling protein 1, UCP1, and the upregulation of other thermogenesis-related genes in ApoA5^-/-^ hamsters. Interestingly, although thermogenesis was increased, their body temperature did not show a significant change, suggesting that circulating FFAs cannot be efficiently released from the LPL-mediated lipolysis of TG-rich lipoproteins in circulation in the absence of ApoA5 and then delivered to adipose tissue for re-esterification and storage. Accordingly, due to a shortage of fatty acids available for thermogenesis, the expression levels of *Atgl* and *Ucp1* were upregulated through a compensatory mechanism to generate heat to maintain their body temperature.

Emerging evidence shows that HTG, especially severe HTG, are tightly associated with systemic inflammation and plasma IL-6 in patients 31. In chow diet-fed ApoA5^-/-^ hamsters, a low-grade inflammatory status was observed in the body. IL-6 protein level was increased in BAT; however, the mRNA expression of *Il-6* remained unaltered. Thus, it is possible that IL-6 was originated from other organs to execute its function, such as liver-adipose crosstalk. In addition, the nuclear transcriptional factor NR1D1 has been shown to suppress IL-6 expression and inflammation 24. Consistently, by combining transcriptomics to analyze the pathogenesis underlying fatty liver, we identified the involvement of the nuclear transcription factor NR1D1 that inhibits lipid synthesis, inflammation, and fibrosis. It was rational for us to propose that ApoA5 regulates fatty liver progression by modulating NR1D1. Our mechanistic study showed that inactivation of ApoA5 reduced the levels of both NR1D1 mRNA and protein by impairing the mRNA stability of NR1D1, thus providing the evidence that NR1D1 plays the essential roles in modulating lipid metabolism and inflammation in the context of ApoA5 deficiency.

Since the relationship between ApoA5 and NR1D1 had been documented in the present study, we next perform diverse experiments to evaluate the efficacy of therapeutic approach. As expected, human NR1D1 overexpression or human ApoA5 restoration by AAV8 vector for 4 weeks improved hepatic lipid accumulation in ApoA5^-/-^ hamsters without or with affecting plasma lipid levels, respectively. This difference in the outcome probably can be explained the possibility that NR1D1 overexpression did not alter ApoA5 expression, but inhibited hepatic lipid synthesis and inflammation; however, ApoA5 restoration not only increased NR1D1 levels, but also activated LPL-mediated lipolysis to reduce plasma lipid levels. These findings also help to clarify the mechanism by which ApoA5 regulates NR1D1. An investigation led by Sharma et al reported that injection of hApoA5 into ApoA5 knockout mice indeed significantly reduced plasma TG levels on chow diet, but unfortunately, there was no change in the hepatic TG contents between null-AAV and hApoA5-AAV groups 17, which suggested that targeting ApoA5 in other animal species should be considered to better understand the relationship between ApoA5 deficiency and NAFLD. However, it was important to note that AAV8-mediated overexpression of human ApoA5 did not fully restore plasma triglyceride levels. Moreover, overexpression of both NR1D1 and ApoA5 via AAV8 did not reverse severe hyperlipidemia and NASH in ApoA5^-/-^ hamsters and fed an HFD (data not shown), raising a concern of gene therapy applied to patients with severe HTG due to ApoA5.

Considering the limitation of gene therapy in our study, we sought to develop an efficient and safe therapeutic method to treat the hamsters lacking ApoA5. Since cold exposure or pharmaceutical therapy to activate BAT by has been reported to increase circulating IL-6 and play beneficial roles in lipid metabolism and NAFLD by enhancing thermogenesis and increasing energy expenditure 28. Surprisingly, exposure to cold or subcutaneous injection of the β3-AR agonist CL316243 completely corrected severe HTG and the pathogenesis of liver in ApoA5^-/-^ hamsters under both chow diet and HFD feeding conditions. This was probably attributed to an enhanced consumption of large amounts of lipids to counteract the cold, leads to significant improvement in hyperlipidemia and reduction in lipid accumulation in liver, implying that the activation of fat tissue through cold exposure or CL316243 may offer superior treatment options.

Elevated plasma TG level caused by ApoA5 deficiency has been considered an independent risk factor of NAFLD; however, the relationship between ApoA5 and ASCVD remains unknow, and thus whether in the setting of ApoA5 deficiency, NAFLD with TG accumulation in the liver promotes the development of has not been well documented. Previous study using murine models showed that overexpression of ApoA5 in mice lacking ApoE exhibited reduced plasma lipid levels, then leading to reduced atherosclerotic lesions relative to control animals 32, but unfortunately, the characteristics of atherogenesis had not been reported yet in the any knockout animals 33. Given that most of variants of ApoA5 genes are loss-of-function mutations in clinical patients, which result in a significant reduction in ApoA5 level to the different extents 34, 35, it is crucial to investigate the role of ApoA5 deficiency in atherogenesis in our hamster model. Although depletion of ApoA5 predisposed to HTG, unexpectedly, atherosclerotic lesions were not observed in young (8-month old) and aged (18-month old) ApoA5^-/-^ hamsters on chow diet and only HFD-fed knockout hamsters exhibited mild atherosclerosis relative to the WT animals. These findings are distinct from the previous observations in hamsters lacking ApoC2, a model showing HTG, in which spontaneous atherosclerotic lesions were found at the age of 8 months without any nutrient intervention 36. This discrepancy could be attributable to the different plasma lipoprotein profiles modulated by LPL activity. In ApoC2^-/-^ hamsters under chow diet condition, plasma LPL activity was completely abolished in the absence of its obligatory cofactor ApoC2 and circulating TG concentration was incredibly increased to 8000-10000 mg/dL, accompanied by hypercholesterolemia with TC level near to 1000 mg/dL, whereas with the same nutrient treatment, ApoA5^-/-^ hamsters showed a reduction in LPL activity by 50%, which was consistent with the observations in ApoA5-deficient patients^37^, leading to HTG with TG level at 2000-3000 mg/dL and a moderate increase in TC level to 200-300 mg/dL. We speculated that compared with the severe combined hyperlipidemia in ApoC2^-/-^ hamsters, the relatively lower lipid levels were not sufficient to initiate the visible atherosclerotic lesions in ApoA5^-/-^ hamsters. Interestingly, it is of note that HFD intervention caused more severe combined hyperlipidemia in ApoA5^-/-^ hamsters, but only mild atherosclerotic development was shown. This unreported finding can be explained by the possibility that the detrimental lipids, including cholesterol and other DAG subclasses, were mostly incorporated into very large TG-rich lipoprotein particles in circulation, which could not enter the subendothelial space to trigger atherogenesis; however, Under HFD condition, our transcriptome data demonstrated that the changes in top 30 functional genes regulated by NR1D1 were shifted from lipid metabolism to inflammatory response, indicating that inflammation, rather than severe combined hyperlipidemia, became a major contributor to the pathogenesis of atherosclerosis. Therefore, to exclude the interference of very large TG rich lipoproteins, it will be tempting for us to further validate whether ApoA5 deficiency can cause an increased incidence of ASCVD by crossing ApoA5^-/-^ hamsters with LDLR-deficient background or by overexpressing PCSK9 in liver in the future study.

In summary, we successfully generated an ApoA5-deficient hamster model targeted by CRISPR/cas9 gene editing, which showed severe HTG with low-grade inflammation, leading to hepatic steatosis under chow diet condition. Loss of ApoA5 predisposed hamsters to HFD-induced more severe HTG and NASH with minor effect on atherogenesis by reducing mRNA of NR1D1 and then impairing NR1D1-mediated metabolic pathways. Gene therapy using AAV8-mediated NR1D1 overexpression or ApoA5 restoration significantly attenuated hepatic steatosis in ApoA5^-/-^ hamsters with or without affecting plasma lipid profiling, respectively, whereas activation of adipose tissue by exposure to cold or CL316243 completely ameliorated hyperlipidemia and hepatic steatosis due to ApoA5 deficiency. Our findings suggest that ApoA5 plays a protective role in lipid disorder and NAFLD, and provide a new insight into the development of therapeutic approaches to treat ApoA5-deficient patients with HTG and NAFLD.

## Methods

### Generation of ApoA5 knockout (ApoA5^-/-^) hamster model

Using Optimized CRISPR Design (http://crispr.mit.edu/), the sgRNA was designed to target the exon 2 of ApoA5 gene (NW_024429203) in Syrian golden hamster. The specificity of target sequences (GCAACCACTCAGGCGCGGAA) was analyzed according to the NCBI BLAST applied to the genome of Syrian golden hamster. The DNA template of sgRNA was amplified by PCR, and then transcribed to sgRNA (100 ng/μL) by T7 polymerase (Megascript T7 Kit, Ambion, AMB13345) *in vitro*. Cas9 mRNA was prepared as described previously. The plasmid PXT7 carrying Cas9 cDNA was used as the DNA template, then linearized by XbaI and purified by ethanol precipitation. Cas9 mRNA (500 ng/μL) was transcribed by linear template using mMESSAGE mMACHINE T7 kit (Ambion, AM1344), and purified by isopropanol precipitation after phenol chloroform extraction. Cas9 mRNA and sgRNA were stored at -80 °C for the future experiments. Both microinjection and zygote treatment were performed under microscope in red light. M2 medium (Sigma, M7167) covered by mineral oil (Sigma, M5904) was used for the injection. Afterward, Cas9 mRNA (50 ng/μL) and sgRNA (20 ng/μL) were co-injected into the cytoplasm of zygotes and then the zygotes were cultured in HECM-10 medium with 10% CO_2_ at 37.5 °C for 30 min. Subsequently, the injected zygotes with normal morphology were transferred into surrogate hamsters (approximate 15-20 zygotes per oviduct), which were naturally mated with males 1 day before. For genotyping, the genomic DNA extracted from the toes of the founders and their descendants was analyzed by PCR (ApoA5 F: GTGCAGTCTGAGTTCCAGGC, ApoA5 R: TGCTTTGCAGCCATGTAGGG).

### Animals

Wild type (WT) Syrian golden hamsters were purchased from Vital River Laboratory (Beijing, China). ApoA5^-/-^ hamster model was constructed using CRISPR/Cas9 gene editing technology in our laboratory as described above^38^. WT and ApoA5^-/-^ hamsters were housed in a temperature and humidity environment under a 14-hour light/10-hour dark cycle at 24 °C. For all experiments, all animals were matched for age and sex. Male animals were fed a chow diet (CD) (20% protein and 4% fat; Beijing Ke'ao Company, Beijing, China) or a high fat diet (HFD) containing 0.5% cholesterol and 20% fat with water ad libitum. WT and ApoA5^-/-^ hamsters were injected with AAV8-hApoA5, AAV8-hNR1D1 or AAV8-GFP via the intrajugular vein at a dose of 1×10^14^ vg/kg. The β3-AR agonist CL316243 was given at a dose of 600 μg/kg/day by subcutaneous injection for 4 weeks. At the endpoint of the experiments, animals were anesthetized with 3% pentobarbital sodium (45 mg/kg by intraperitoneal injection). All experiments were performed under the principle of experimental animal care (NIH publication no.85Y23, revised 1996) and were approved by the laboratory animal ethics committee of Peking University (LA2022147).

### Plasma biochemical characteristics

Plasma samples were collected from the retro-orbital plexus of the hamsters after 12-hour fasting. Free fatty acid (FFA) was measured with commercially enzymatic kit (633-52001, Wako, Japan). Plasma glucose was determined with commercially enzymatic kits (Biosino Bio Technology & Science, Beijing, China). The degree of liver injury was estimated based on the plasma alanine aminotransferase (ALT) and aspartate transaminase (AST) levels (Nanjing Jiancheng Bioengineering Institute, Nanjing, China). The plasma MDA concentration was measured using a commercial test kit (E2009, Applygen, China). Briefly, a 100 μL fresh plasma sample or serially diluted standard samples were thoroughly mixed with 300 μL buffer containing sodium dodecyl sulfate and thiobarbituric. The mixture was incubated at 95 °C for 30 min and then placed on ice for 5 min, followed by a centrifugation at 10, 000 g for 10 min. 200 μL supernatant was collected for fluorometric measurement using ex535 nm/em553 nm, which was converted to the concentrations according to the standard curve.

### Analysis of plasma lipids and lipoproteins

Plasma was collected from WT and ApoA5^-/-^ hamsters after 12 h fasting. Plasma total cholesterol (TC) and triglyceride (TG) levels were determined with commercially enzymatic kits (Biosino Bio Technology & Science, Beijing, China). HDL-cholesterol (HDL-C) level was measured by the same TC kit after precipitating ApoB-containing lipoprotein by 20% polyethylene glycol (PEG).

Plasma ApoB, ApoE and ApoA1 were detected by Western blots. In Brief, 1μL plasma was mixed with the 5× SDS loading buffer and heated at 95 °C for 10 min. The samples were loaded to 6% or 12% sodium dodecyl sulfate polyacrylamide gel (SDS-PAGE) gels for ApoB or ApoE/ApoA1, respectively. The following antibodies were used: ApoB (178467, Millipore, goat polyclonal IgG, 1:2000), ApoE (178479, Millipore, goat polyclonal IgG, 1:2000) and ApoA1 (ab20453, Abcam, rabbit polyclonal IgG, 1:2000).

For the plasma apolipoprotein analysis of HFD-fed animals: lipids were depleted by ether/methanol from the isolated total lipoprotein particles, which were then subjected to Tricine-SDS- PAGE gel and visualized by Coomassie brilliant blue staining.

To analyze the lipid distribution, pooled plasma aliquots from 6 to 8 hamsters per group (100 μL/group after filtered by 0.22-mm filter) were applied to fast protein liquid chromatography (FPLC, Tricorn high-performance Superose S-6 10/300GL column). The samples were eluted with PBS at a constant flow rate at 0.5 mL/min. The TC and TG levels of the eluted fractions (500 μL per fraction) were measured using the same kits as described above. Three continuous fractions were mixed together with the buffer containing SDS and DTT, and heated at 95 °C for 10 min. The contents of ApoA1, ApoB and ApoE were detected by Western blots according to the method described above.

### Metabolic assays

#### Oral fat load

In the oral fat load assay, WT and ApoA5^-/-^ hamsters were fasted 12 h, then gavaged with olive oil (10 mL/kg body weight). Plasma was collected at the indicated time points (0 h, 0.5 h, 1 h, 2 h, 4 h and 8 h) after gavage for TG measurement.

#### VLDL secretion assay

After fasting for 12 h, plasma was collected from indicated groups on CD feeding, which represented a basal value at 0 min. Afterward, hamsters were intraperitoneally injected with 7.5% Poloxamer 407 (CAS 9003-11-6, Sigma, 1500 mg/kg), and blood samples were collected at 30, 60, 120, 180 and 240 min after injection. TG concentration was measured using enzymatic methods for each indicated time point. After linear regression, the slope represented VLDL secretion rate.

#### LPL activity assay

Plasma was collected 30 min after intraperitoneal injection of heparin (2000 U/kg body weight). Plasma LPL activity was analyzed using a commercial LPL activity kit (ab204721, Abcam, USA) containing fluorescence labeled substrates and LPL activator. LPL activity was present by free fatty acids (FFA) release (pmol/mL/min).

### Glucose tolerance test (GTT)

After fasting for 12 h, plasma was collected from indicated groups on CD feeding, which represented a basal value at 0 min. Afterward, hamsters were intraperitoneally injected with glucose solution (2 g glucose/kg body weight), and blood samples were collected at 15, 30, 60 and 120 min after injection. Glucose levels were measured using enzymatic methods for each indicated time point.

### Insulin tolerance test (ITT)

After fasting for 6 h, plasma was collected from indicated groups on CD feeding, which represented a basal value at 0 min. Afterward, hamsters were intraperitoneally injected with insulin (0.75 U insulin/kg body weight), and blood samples were collected at 15, 30, 60, and 120 min after injection. Glucose levels were measured using enzymatic methods for each indicated time point.

### Pathological analysis

Hamsters at the indicated time points under different conditions were sacrificed and perfused with 0.01 M cold PBS through the left ventricle. Liver, intestine, white adipose tissue (WAT), brown adipose tissue (BAT), heart, and aorta were harvested and then fixed in 4% paraformaldehyde (PFA) overnight, followed by the dehydration using 20% sucrose solution. Then liver, intestine and heart were embedded in SAKURA Tissue-Tek® O.C.T. Compound (Sakura Finetek USA, Inc., USA), and cryo-sectioned ​​after snap frozen with liquid nitrogen. Sections were stained with hematoxylin/eosin (HE) for the morphological analysis, oil red O (ORO) for lipid deposition analysis and sirius red for fibrosis analysis. WAT and BAT were embedded in paraffin and sliced for HE staining.

To measure the expression levels of specific proteins in tissues, the following antibodies were used for immunohistochemical or immunofluorescence staining. Immunohistochemical staining was performed with UCP1 antibody (1:100 rabbit polyclonal IgG, A5857, ABclonal, China) in BAT and WAT. Immunofluorescence staining was performed with CD68 antibody (1:200 rabbit polyclonal IgG, BM3639, BOSTER, China) for macrophage in liver, TH (Tyrosine hydroxylase) antibody (1:200 rabbit polyclonal IgG, A12756, ABclonal, China) in WAT and IL-6 (interleukin 6) antibody (1:100 rabbit polyclonal IgG, BA4339, BOSTER, China) in BAT.

### Hepatic cholesterol/triglyceride contents

100 mg of liver tissue was homogenized in 1 mL cold phosphate buffer solution (PBS), and then 4 mL of chloroform/methanol (v:v = 2:1) was added. The mixture was vortexed for 2 min, and then kept on ice for 20 min. After a centrifugation at 3000 rpm for 30 min, the chloroform layer was transferred to a new glass tube with a glass syringe and dried under nitrogen stream. The contents of cholesterol and triglyceride were measured by dissolving lipids with 500 μL 3% triton X-100.

### RNA isolation and quantitative real time PCR

Total RNA was extracted from different tissues or cells by Trizol reagent (Transgen Biotech, China) and first-strand cDNA was reversely transcribed using a RT kit (Transgen Biotech, China). Quantitative real time PCR was performed using primers listed in supplement table. Amplification reactions were performed using the Mx3000 Multiplex Quantitative PCR System (40 cycles: denaturation at 94 °C for 15 s, annealing at 60 °C for 20 s, and extension at 72 °C for 45 s. β-actin was used as internal control and all the data of gene expression were normalized to WT group. Quantitative real time PCR was performed using primers listed in Table S1.

### Western blot analysis

Tissues and cells were lysed with cold RIPA lysis buffer (DiNing, DN105-01, China) containing a protease inhibitor (04693132001; Roche, Basel, BS, Switzerland) and phosphatase inhibitor (4906837001; Roche, Basel, BS, Switzerland). Total protein was extracted by centrifugation and quantified with a BCA kit (23225; Thermo, Waltham, MA, USA). Samples containing equal quantities of protein were separated by 10% SDS-PAGE, transferred to NC membranes (66485, Pall Bio Trace, USA), blocked with 5% non-fat milk in TBST, incubated with the indicated primary antibodies overnight at 4 °C, then followed by the appropriate HRP-conjugated secondary antibodies. Finally, signals were detected with an ECL kit (Transgene, China) and visualized in Invitrogen iBright1500 Imaging System (Thermo Fisher, USA). The antibodies used for our experiments include the customized hamster ApoA5 antibody (1:1000, rabbit polyclonal IgG, Sino Biological, China), the ApoA5 antibody (1:1000, mouse polyclonal IgG, Santa Cruz, USA), the NR1D1 antibody (1:1000, rabbit polyclonal IgG, Abclonal, China), the β-actin antibody (1:1000, mouse polyclonal IgG, Abclonal, China), the GAPDH antibody (1:1000, mouse polyclonal IgG, Abclonal, China), the Lamin B1 antibody (1:1000, rabbit polyclonal IgG, Abclonal, China).The arbitrary densitometry units of the proteins were quantified by ImageJ image software and expressed as means ± SEM. The results were presented by the ratio of the values normalized to WT group.

### Coimmunoprecipitation (co-IP) assay

For coimmunoprecipitation (co-IP) assays, hamster liver tissues and HepG2 cells were lysed with RIPA lysis buffer containing a protease inhibitor. After centrifugation, the supernatant containing proteins was subjected to IP with antibodies and then indicated protein G agarose beads overnight at 4 °C. The beads were washed with NaCl buffer and boiled with 2× SDS loading buffer prior to analysis by Western blots.

### Chromatin immunoprecipitation (ChIP) assay

For Chromatin immunoprecipitation assays, HepG2 cells were fixed with formaldehyde, and cross-linked protein-DNA complexes were prepared using a ChIP kit (P2083, Beyotime). Complexes were incubated with a primary antibody against ApoA5 or rabbit IgG overnight at 4 °C with gentle agitation. Magnetic beads were added with gentle agitation for 1 h. DNA was eluted from the beads. Primers on the human NR1D1 promoter were used in qPCR assays, and PCR results were visualized using agarose gel electrophoresis.

Primer1F: ACTCTCACCCTGTTGCCCAG, R: CCTGTAGTCCCAGCCACT, Primer2F: ATTAGCGGGAGCAGCAGGTG, R: CGCTTCAGAGCAGCAACTGT, Primer3F: CCGGCAATGCTGGCTGTTT, R: GAAGTGACCGACCCAAGGTCA, Primer4F: GGAGTGGGTGGAATGGTGTCA, R: CCTCTGCTTCAGGGCAAAGTCC, Primer5F: CACATGGTACCTGCTCCAGT, R: GCTACGTTCCCTCGGCAGTA.

### RNA pulldown assay

For biotin pull-down assays, biotinylated RNA was transcribed from PCR-amplified DNA using T7 RNA polymerase in the presence of biotin-UTP. One microgram of purified biotinylated transcripts was incubated with 100 µg of cytoplasmic extracts for 30 min at room temperature. Complexes were isolated using paramagnetic streptavidin-conjugated Dynabeads (Dynal, Oslo). The pull-down material was analyzed by Western blotting.

As a template, cDNA was used for PCR amplification of RNA fragments. All 5′ primers contained the T7 promoter sequence (TAATACGACTCACTATAGG). And we used following primer pairs to prepare templates for the *Nr1d1* mRNA fragments:

(T7)AGAGTGAAATATTACTGCCGAGGGAAC and GTCTTCACCAGCTGAGAGCG for 5' UTR, (T7)ATGACGACCCTGGACTCCAA and TGCCCACAGAGAGACACTT for CR1, (T7)TGTCTCGAGACGCTGTGC and GGACATGCCAGCAGAACATT for CR2, (T7)TATGAACATGTACCCGCAT and CTGGGCGTCCACCCGGAAGGACA for CR3 and (T7)AAGCTGCTGTCCTTCCGGGT and TCAGCTGTGAACTATTGGATTTGAGACAG for 3'UTR.

### Cell culture and *in vitro* treatment

Hamster primary hepatocytes were isolated from 4-week-old male WT or ApoA5^-/-^ hamsters using a collagenase perfusion and gradient centrifugation method. Hamsters were anesthetized and perfused via the portal vein with perfusion medium (100 mL D-hanks + 0.02 g EDTA) followed by digestion medium (100 mL D-hanks + 0.056 g CaCl_2_ + 0.238 g HEPES + 0.003 g collagenase IV). Then, the livers were excised and filtered through a 100 μm cell strainer. Hepatocytes were separated via centrifugation at 500 g and were then cultured with Dulbecco's modified Eagle's medium (DMEM) containing 10% FBS and 1% penicillin-streptomycin at 37 °C.

HepG2 cells were cultured with DMEM supplemented with 10% FBS (FS401, Transgene, China) and 1% penicillin-streptomycin (15140-122, Gibco by Invitrogen, USA) in a 37 °C, 5% CO_2_ incubator. ApoA5 siRNA was transfected into HepG2 cells with Lipofectamine RNAiMAX (Invitrogen, 13778150, Thermo Fish, USA) to knockdown ApoA5, and plasmid expressing human NR1D1 was transfected into HepG2 cells with Hieff Trans® Liposomal Transfection Reagent (40802ES03, Yeasen, China) to overexpress human NR1D1. HepG2 cells were exposed to the culture medium containing BSA conjugated-palmitic acid (500 μM) (PA; P0500; Sigma-Aldrich, USA) for 12-16 hours. Fatty acid-free BSA (0332, Amresco, USA) was used as a control. After treated with PA, HepG2 cells were fixed with 4% paraformaldehyde and stained with 60% Oil red O solution (O1391; diluted with water; Sigma-Aldrich, USA) for 30 min. To analyze the half-lives of *nr1d1* mRNAs, actinomycin D (ActD, final concentration 2 μg/mL, Bioss, D50409s, Beijing, China) was added to hamster primary hepatocyte and HepG2 cell cultures. Forty-eight hours after siRNA transfection, the total RNAs were extracted at 0, 2, 4 and 6 h after ActD treatment. The mRNA levels at different times were analyzed by using RT-qPCR.

### Lipidomics analysis

Lipidomic analysis was conducted at LipidALL Technologies using a Shimadzu Nexera 20-AD/ExionLC-AD coupled with Sciex QTRAP 6500 PLUS as reported previously 39. The polar lipids were separated by UP-Hb silica gel column (i.d. 150x2.1 mm, 3 μm) and normal phase (NP)-HPLC method. The mobile phase A was chloroform: methanol: ammonium hydroxide at the ratio of 89.5:10:0.5, and the mobile phase B was Chloroform: methanol: ammonium hydroxide: water at the ratio of 55:39:0.5:5.5. MRM targeted quantitative technique was established for comparative analysis of various polar lipids, and lipids were quantified by adding internal standard. Specific lipidomics analysis were performed by Novogene Co., LTD (Beijing, China).

### RNA-seq analysis

Total RNA was extracted using TRIzol reagent (ET111-01-V2; Transgene, Beijing, China) and the RNA integrity was measured using the Agilent 2100 bioanalyzer system. Libraries of amplified RNA were prepared in accordance with the Illumina protocol. Then, gene expression profiling was performed in TruSeq PE Cluster Kit v3-cBot-HS (Illumia) by sequencing on an Illumina Novaseq platform, and 150 bp paired-end reads were generated. The reads were mapped to Mesocricetus auratus reference genomes with HISAT2 software. The Fragments Per Kilobase of exon model per Million mapped fragments (FPKM) value of each identified gene was calculated with StringTie. Differentially expressed genes (DEGs) were identified by differential gene expression analysis conducted with DESeq2. Preparation of RNA library and transcriptome sequencing was conducted by Novogene Co., LTD (Beijing, China). Genes with adjusted p-value < 0.05 and |log2(FoldChange)| > 0 were considered as differentially expressed. The clusterProfiler R package was used to perform Gene Ontology (GO) enrichment analysis of DEGs. The GO terms with padj less than 0.05 were considered significantly enriched by DEGs.

### Cold Exposure

Hamsters were individually housed with ad libitum access to food and water during exposure to 4 °C in a cold room with a 12-h light-dark cycle for 5 days in chow diet condition. HFD-fed hamsters for 4 weeks were exposed to intermittent 6 h/d cold exposure for 7 days. After cold exposure 12 h-fasted plasma lipids were determined as described above. Then hamsters at the indicated time points under different conditions were sacrificed and perfused with 0.01 M cold PBS through the left ventricle.

### Statistical analysis

All data were presented as means ± SEM. GraphPad Prism 8.0 software was used for all statistical analysis. The analysis was performed using the Student's t-test for the comparison between two groups and one-way ANOVA for the comparison among multiple groups. P value less than 0.05 was considered statistically significant.

## Supplementary Material

Supplementary figures and tables.
