# Supplementary material for: Depletion of ApoA5 aggravates spontaneous and diet-induced nonalcoholic fatty liver disease by reducing hepatic NR1D1 in hamsters
Source: Theranostics. 2024 Feb 24;14(5):2036–57. doi: 10.7150/thno.91084 (PMC10945338; doi:10.7150/thno.91084)
Supplement: Supplementary file 1 — Supplementary figures and tables. [file thnov14p2036s1.pdf]

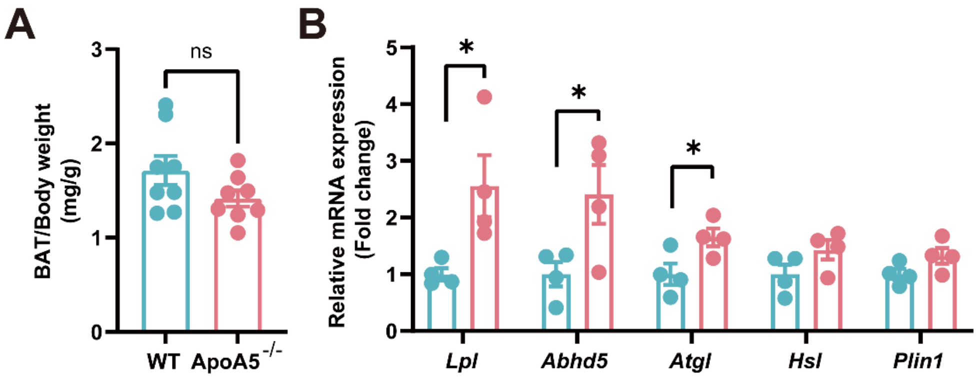

Figure S1. Analysis of the ratio of BAT weight/body weight and the mRNA expression of genes regulating lipolysis in BAT in WT ApoA5$^{-/-}$ hamsters on chow diet.

A: The ratio of BAT weight/body weight from 3-month-old male WT and ApoA5$^{-/-}$ hamsters on chow diet (n = 8/group).

B: The expression levels of genes involved in lipolysis in BAT were determined by real-time PCR (n = 4/group). Error bars represent mean ± SEM. *P < 0.05; **P < 0.01; ***P < 0.001; ns, not significant.

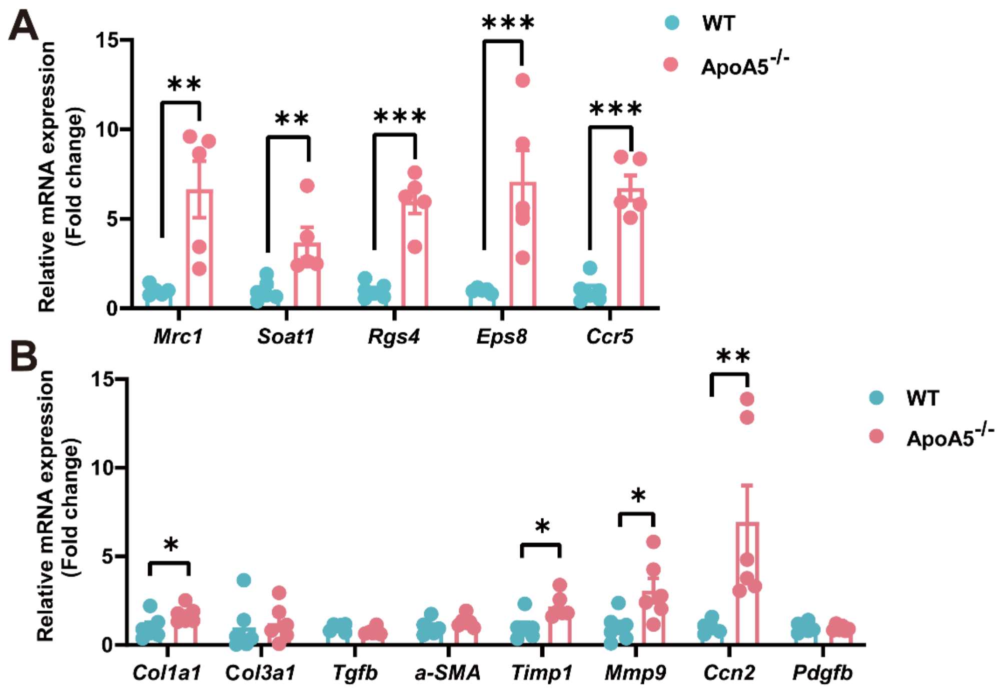

Figure S2. Alterations in the mRNA expression of the inflammatory and fibrotic genes caused by ApoA5 deficiency under HFD condition.

A: The expression levels of genes involved in inflammation in the livers of HFD-fed WT and ApoA5$^{-/-}$ hamsters for 12 weeks were determined by real-time PCR (n = 5-6/group).

B: The expression levels of genes involved in fibrosis in in the livers of HFD-fed WT and ApoA5$^{-/-}$ hamsters for 12 weeks were determined by real-time PCR (n = 6/group).

Error bars represent mean ± SEM. *P < 0.05; **P < 0.01; ***P < 0.001; ns, not significant.

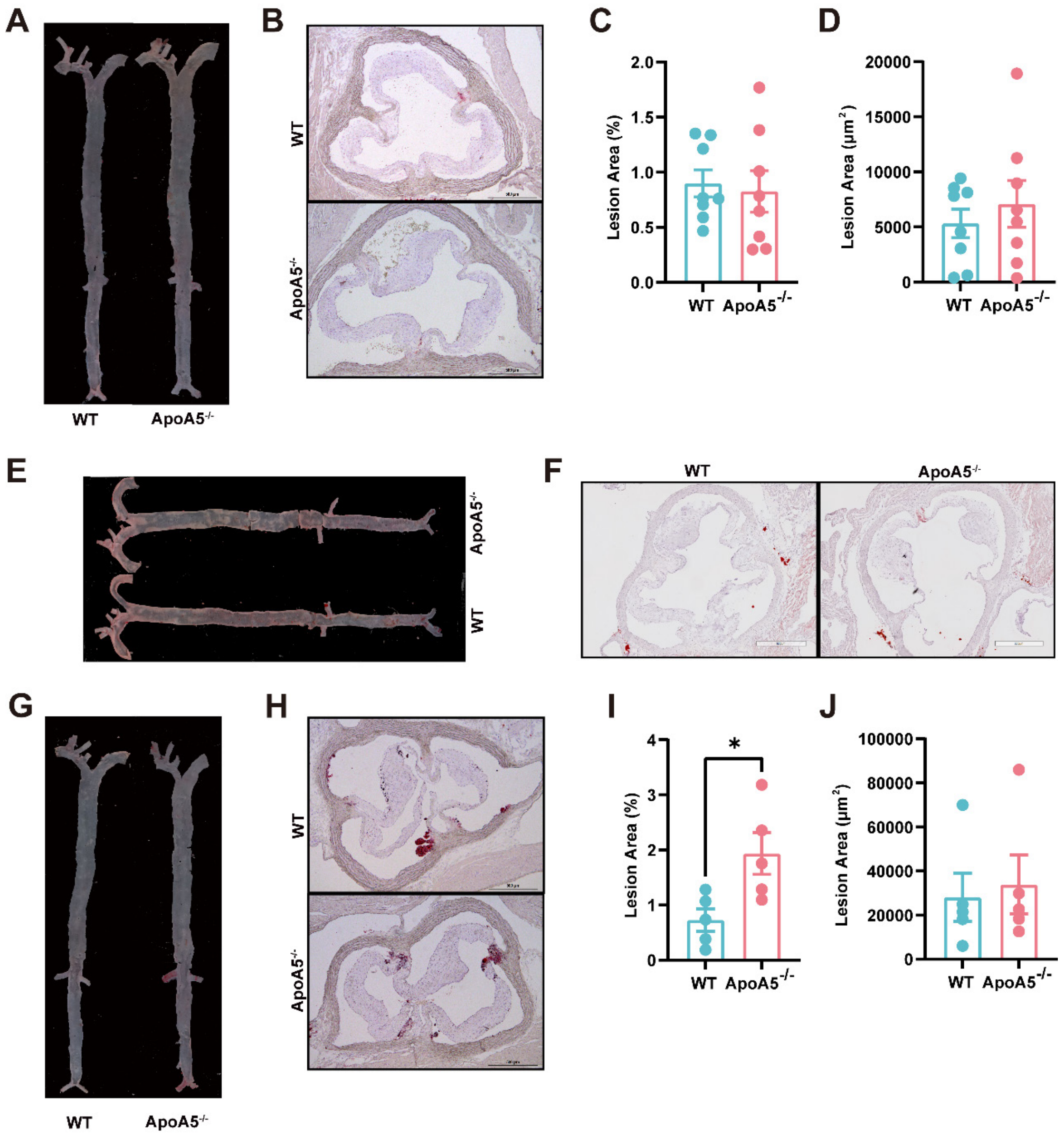

Figure S3. ApoA5 inactivation has mild effect on spontaneous atherosclerosis under chow diet and HFD conditions.

A-D: Analysis of atherosclerotic lesions in whole aorta (A, C) and sectioned aortic roots (B, D) of 8-month-old WT and ApoA5$^{-/-}$ hamsters (n = 8/group).

E-F: Analysis of atherosclerotic lesions in whole aorta (E) and sectioned aortic roots (F) of 18-month-old WT and ApoA5$^{-/-}$ hamsters (n = 6/group).

G-J: Analysis of atherosclerotic lesions in whole aorta (G, I) and sectioned aortic roots (H, J) of HFD-fed WT and ApoA5$^{-/-}$ hamsters for 12 weeks (n = 5/group). Error bars

represent mean ± SEM. *P < 0.05; **P < 0.01; ***P < 0.001; ns, not significant.

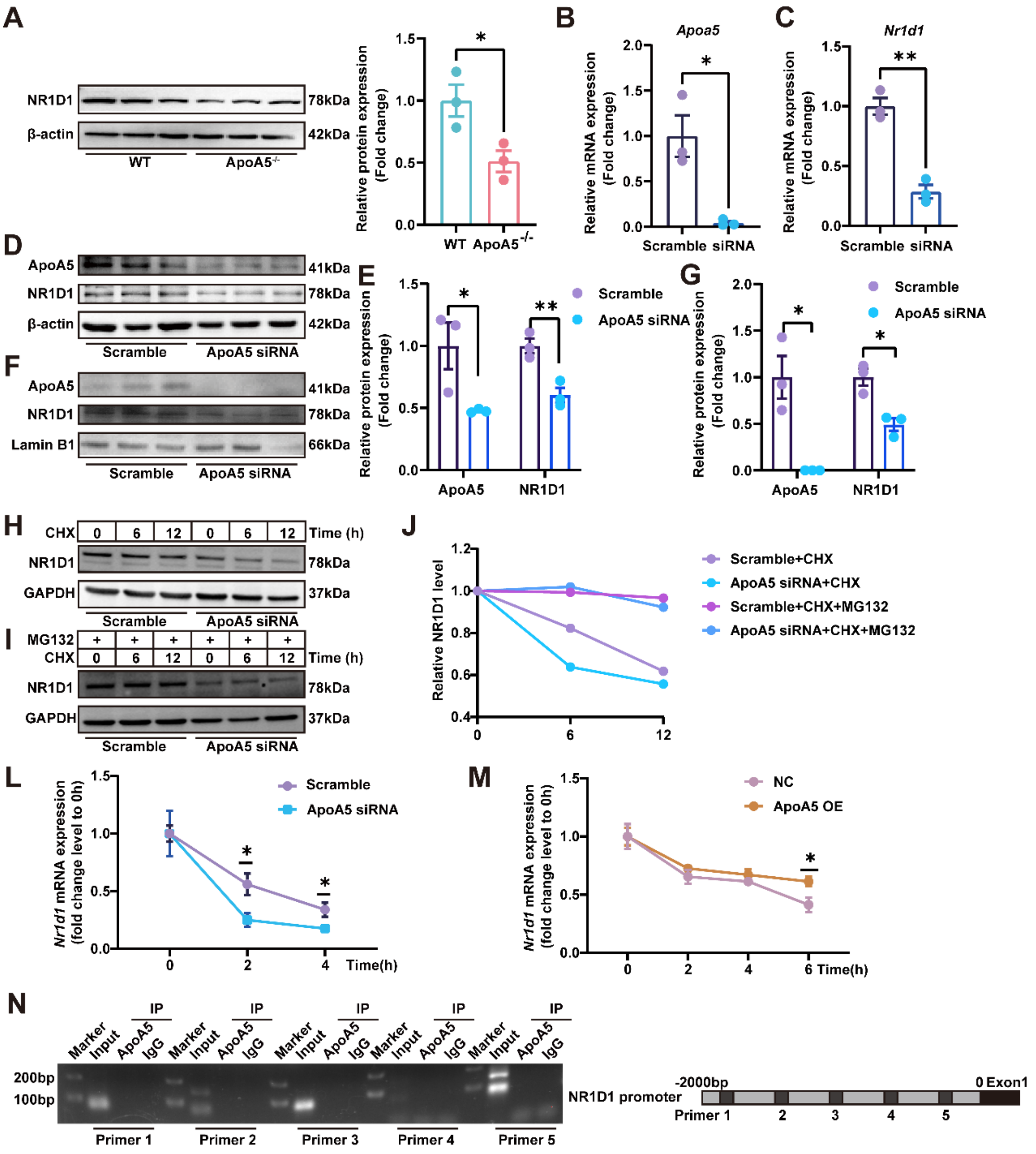

Figure S4. Validation of the relationship between ApoA5 and NR1D1 in HepG2 cells

A: Western blot analysis of NR1D1 protein in the liver samples of HFD-fed WT and ApoA5$^{-/-}$ hamsters and quantitative data (n = 3/group).

B: The mRNA expression levels of Apoa5 in HepG2 cells transfected with scramble and ApoA5 siRNA were determined by real-time PCR (n = 3/group).

C: The mRNA expression levels of *Nr1d1* in HepG2 cells described in (A) (n = 3/group).

D-E: Western blot analysis of NR1D1 protein levels in HepG2 cells described in (B) and quantitative data (n = 3/group).

F-G: Western blot analysis of nuclear ApoA5 and NR1D1 protein levels in HepG2 cells described in (B) and quantitative data (n = 3/group).

H-J: HepG2 cells were transfected with scramble or ApoA5 siRNA and treated with CHX (50 μg/mL) and MG132 (10 μM) for 0, 6 and 12 hours. The relative NR1D1 protein levels were quantified.

L: The mRNA levels of *Nr1d1* of scramble or ApoA5 siRNA transfected HepG2 cells treated with Actinomycin D (2 μg/mL) of HepG2 cells (n = 3/group).

M: The mRNA levels of *Nr1d1* of NC (negative control) or ApoA5 plasmid transfected HepG2 cells treated with Actinomycin D (2 μg/mL) of HepG2 cells (n = 3/group).

N: ChIP assays were performed by using HepG2 cell lysates and antibodies against ApoA5 and IgG. Error bars represent mean ± SEM. *P < 0.05; **P < 0.01; ***P < 0.001; ns, not significant.

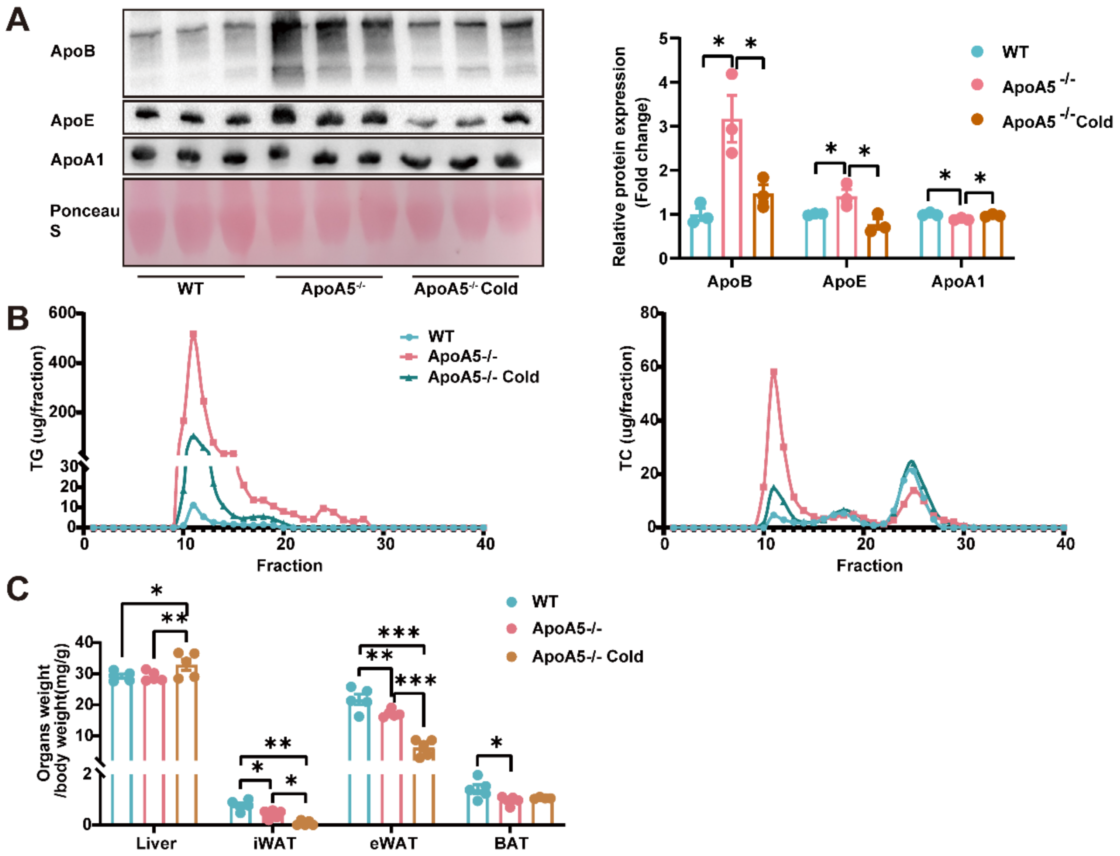

Figure S5. The changes in lipid profiling and the ratio of organ weight/body weight in CD-fed ApoA5$^{-/-}$ hamsters exposed to the cold treatment.

A: Representative Western blots of plasma ApoB, ApoE and ApoA1 from 3-month-old male WT and ApoA5$^{-/-}$ hamsters with or without cold exposure for 5 days and quantitative data (n = 3/group).

B: Pooled plasma from the three groups were analyzed by FPLC. TG and TC contents in different fractions of pooled plasma from the animals described in (A) were measured (n = 4-5/group).

C: The ratio of Liver/WAT/BAT weight and body weight from the animals described in (A) (n = 4-5/group). Error bars represent mean ± SEM. $*P < 0.05$; $**P < 0.01$; $***P < 0.001$; ns, not significant.

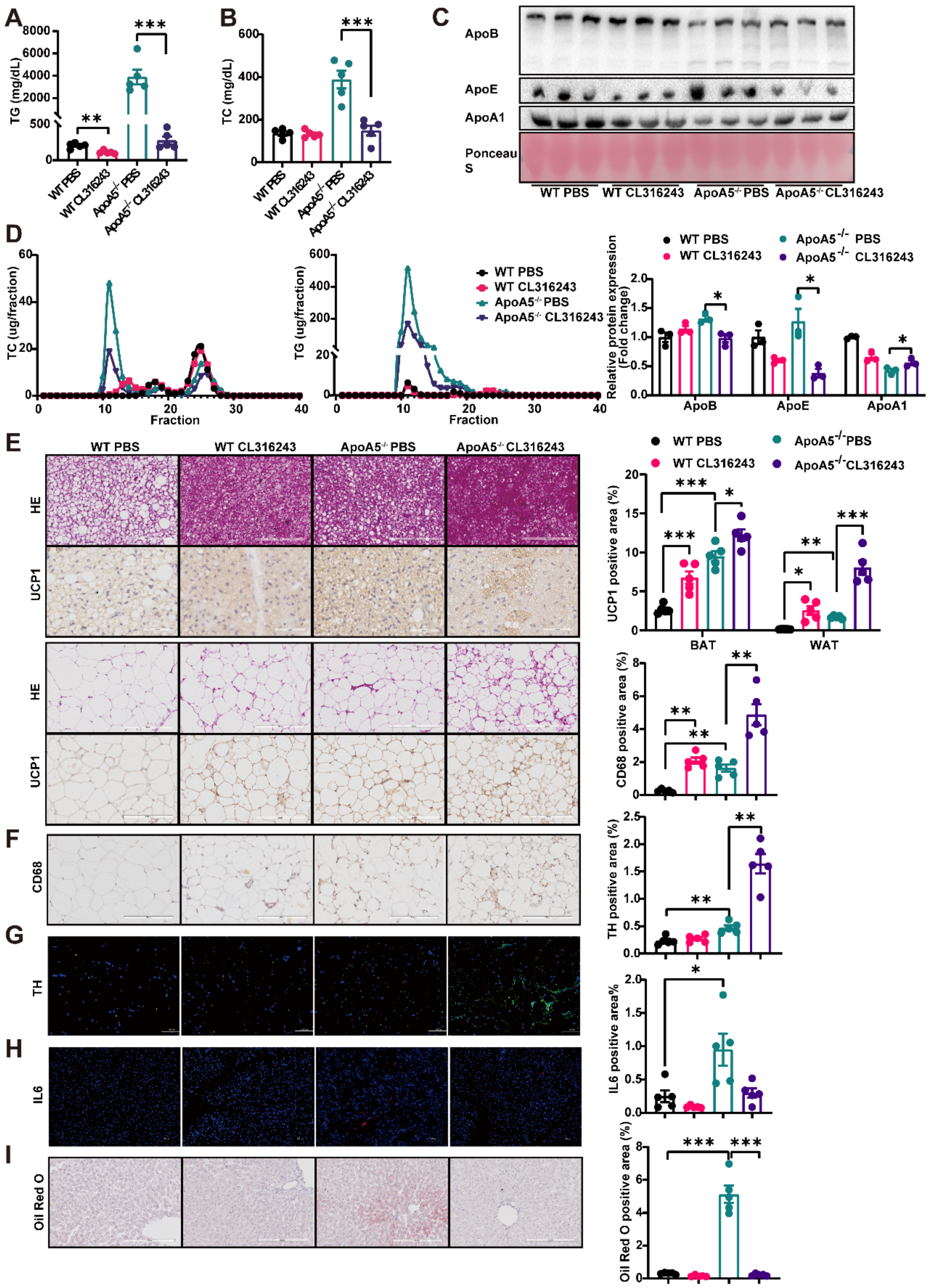

Figure S6. Activation of adipose tissue by CL316243 ameliorated lipid metabolism disorders and hepatic steatosis caused by ApoA5 deficiency

A-B: Plasma triglycerides (D) and total cholesterol (E) determined from WT and $ApoA5^{-/-}$ hamsters treated with CL316243 4 weeks (n = 5/group).

C: Representative Western blots of plasma ApoB, ApoE and ApoA1 from WT and $ApoA5^{-/-}$ hamsters treated with CL316243 for 4 weeks and quantitative data (n = 3/group).

D: Pooled plasma from the three groups were analyzed by FPLC. Triglyceride and cholesterol contents in different fractions of pooled plasma from WT and $ApoA5^{-/-}$ hamsters treated with CL316243 for 4 weeks were measured (n = 5/group).

E: The representative images of HE and UCP1 immunohistochemical staining in BAT and eWAT sections of WT and $ApoA5^{-/-}$ hamsters treated with CL316243 for 4 weeks and quantitative data (n = 5/group).

F: The representative images of CD68 immunohistochemical staining in eWAT sections of WT and $ApoA5^{-/-}$ hamsters treated with CL316243 for 4 weeks and quantitative data (n = 5/group).

G: The representative images of TH immunofluorescence staining in eWAT sections of WT and $ApoA5^{-/-}$ hamsters treated with CL316243 for 4 weeks and quantitative data (n = 5/group).

H: The representative images of IL-6 immunofluorescence staining in BAT sections of WT and $ApoA5^{-/-}$ hamsters treated with CL316243 for 4 weeks and quantitative data (n = 5/group).

I: The representative images of oil red O staining in liver sections of WT and $ApoA5^{-/-}$ hamsters treated with CL316243 for 4 weeks and quantitative data (n = 5/group). Error

bars represent mean ± SEM. *P < 0.05; **P < 0.01; ***P < 0.001; ns, not significant.

Table S1 The list of primers used for quantitative real-time PCR.

Apoa5-F	GCCCACTCTTACTGAAGGCT
Apoa5-R	GCTGCTCTGGCTGAAGTAGT
Apoa1-F	CTGCAGGAGAAGCTAACCCC
Apoa1-R	TTCTTGCTGGCTTCCTCGAC
Apoa4-F	TGACACCCTATGCCAACGAG
Apoa4-R	CCTCCAGGTTCTGGTCGATG
Apoc3-F	TTTCCTTCAGGTGCGTTGGT
Apoc3-R	GAAACCCCAGGCCCAACC
Mttp-F	CAGGTCCAAGAATGGTGCCT
Mttp-R	CTCCGCCAGAGAAGGACATC
Pla2g12b-F	AGACACGTGTGCCTGGAAAT
Pla2g12b-R	CTGGCAACTGAAACATGGGC
Cideb-F	CTGCCGTGGAGAGTGAGGACTT
Cideb-R	GTTCAGGCTGCCGAAGAGGTCT
Sar1b-F	AAGACAAGGCTATGGAGAAG
Sar1b-R	ATTCAAGTTATGCGTGTTGG
Arf1-F	GTGACCACGATTCCCACCAT
Arf1-R	CGCCATAGCGGTCTGATCTT
Sec22b-F	CTGGAAGACCTGCACTCGGAAT
Sec22b-R	CACTACACCTCACAGCCACCAA

Surf4-F	TATTGACACGACCTGGAGCTG
Surf4-R	CTCACGCATGGTTGGAACAC
Lpl-F	TCCTACTTCAGCTGGTCGGA
Lpl-R	CACTTCACAAACACTGCGGG
Abhd5-F	CGGATAGGAGACTTGCACCC
Abhd5-R	TCACGTAGGACTTTGGTCGC
Atgl-F	AAGGAGTGCGCTATGTGGAC
Atgl-R	GATTGCGCAGGTTGAACTGG
Hsl-F	GTTGTCGTCCCTGGCTAACA
Hsl-R	TTCCCGCAGGTCATAGGAGA
Plin1-F	CCCAGCCCTTCAATACCCTC
Plin1-R	TGGTGTGCCGAGAAAGAGTG
Hmgcs1-F	TGGAGGAACTGTCGGTGAGA
Hmgcs1-R	GTTGCAGAGCTAGTCACCGT
Idi1-F	AATTGGGGCTGACACCAAGA
Idi1-R	CTCCGTGCAGCTCGTTTTAC
Fdps-F	CTCCTCTCTCAGAATGAATGGG
Fdps-R	ATTGTACTTGCCTCCTACGGC
Fdft1-F	CACCTACCTGTCAAGGCTCC
Fdft1-R	TTATAACAGGCAGCCAGCGT
Mvk-F	CCAGCAAGGGAAGATGTCGT
Mvk-R	CACTCCAGGGATATGGCGTC

Sc5d-F	AGAATGGTGGCCTCTGCTTC
Sc5d-R	TGGCCTCCTCTACCATCCTC
Sqle-F	TCCGGACCTTTGTGACGATG
Sqle-R	ACCCGTCACACATTCTCCAC
Dhcr7-F	CACTTTGGGTGGTACCTGGG
Dhcr7-R	GCGGAACAGGTCCTTCTGAT
Srebp1c-F	GCGGACGCAGTCTGGG
Srebp1c-R	ATGAGCTGGAGCATGTCTTCAAA
Insig1-F	CTGGTCCTGGGTGTGATGAAG
Insig1-R	AATGTTCCAGTGCAGACAGGT
Acc1-F	ACACTGGCTGGCTGGACAG
Acc1-R	CACACAACTCCCAACATGGTG
Scd1-F	GGAGAAGCAGAAGACCGTTCC
Scd1-R	CCCCTCCTCATCCTGGTAGC
Fasn-F	GCAGTCTTGAGTAGCTTTGTGCT
Fasn-R	GGGAGCTGTCCAGATTAATACCT
Gpam-F	AAATGCAAACCGAAGGTGGC
Gpam-R	GAGGCGCCATTATTTGCAGG
Dgat2-F	ATGAAGACCCTCATCGCTGC
Dgat2-R	CATTCTTGTTCTCGCTGCGG
Nr1d1-F	GGGCTTCTCTCAGTTCCCAC
Nr1d1-R	ACTTGTCATGGGCGTAGGTG

Ucp1-F	GGACAGTTCCTGGTCTACGC
Ucp1-R	CCTCAACAGGTTAGGGGTCG
Cox8b-F	AGTTCCCCAGGCGGCTATAA
Cox8b-R	AGGTTGTGCTCCTTCCTTGG
Cidea-F	GGACAGTTCCTGGTCTACGC
Cidea-R	AAAGGAATGCACCTGGGCTC
Pgc1a-F	TGAATGCAGCGGTCTTAGCA
Pgc1a-R	TTGGAGGCGCATTTGTCTCT
Mrc1-F	GGTGTCGGAATCGCAGGTTA
Mrc1-R	GGCATACAGAGTGACCGAGG
Soat1-F	CGTGACAGCTATCCGAGGAC
Soat1-R	CACACCTGGCAAGATGGAGT
Rgs4-F	GCTCCCCTTCAGTGTTCTCC
Rgs4-R	CAGGCAGGCTCACCATATCA
Eps8-F	CCCAGTGGCTACGGAGTCTA
Eps8-R	CTGTCTCGGGCATAGTGCTT
Ccr5-F	GACACACTGCTGCATCAATCC
Ccr5-R	TGTGGACCGGGTATAGACTG
Col1a1-F	ATGCCGTGACCTCAAGATGTGC
Col1a1-R	TGCTCTCGCCGAACCAGACA
Col3a1-F	GGTCCATCTGGTGACAAGGG
Col3a1-R	GGGTCCAGCTCCTCCTCTAA

Tgfb-F	CAGTTGTACGGCAGTGGCTGAA
Tgfb-R	GTCACGGATGGTGCTCATGTCA
α-SMA-F	CCACCATGTACCCAGGCATT
α-SMA-R	GGCGCTGAACCACAAAACAT
Timp1-F	CCGCAGCGAGGAGTTTCTCATC
Timp1-R	CTGTGGATTCCGTGGCAAGCA
Mmp9-F	CTCTACACGGAGCACGGCAATG
Mmp9-R	AACCATCCGAGCGACCTTCAGT
Ccn2-F	TCTCCAAGCCCGTCAAGTTC
Ccn2-R	GTAATGGCAGGCACAGGTCT
Pdgfb-F	GTGTGGGATGTGTGTTGCAC
Pdgfb-R	GGGCCTCGGAGTGAATTGAA
β-actin-F	ACTGCCGCATCCTCTTCCT
β-actin-R	TCGTTGCCAATGGTGATGAC
Human-Apoa5-F	AGATAGCTGCCTTCACTCGC
Human-Apoa5-R	TTGCTCAGAACCTTGCCACT
Human-nr1d1-F	CGACCCTGGACTCCAACAAC
Human-nr1d1-R	GACTGGAAGCTGCCATTGGA
Human-hdac3-F	AATGCCTTCAACGTAGGCGA
Human-hdac3-R	GGGTTGCTCCTTGCAGAGAT
Human-ncor-F	CAGGTTCTGACAGGCCTCAA
Human-ncor-R	TCATCTCCACATGGTTGCCC

Human-shp-F	TCAAGTCCATTCCGACCAGC
Human-shp-R	AAGAAGGCCAGCGATGTCAA
Human-hmgcr-F	CAGGGAACCTCGGCCTAATG
Human-hmgcr-R	ACAAGCTCCCATCACCAAGG
Human-hmgcs1-F	CGGCTGGAAGTTGGAACAGA
Human-hmgcs1-R	TACCAGGGCATACCGTCCAT
Human-β-actin-F	GCCGCCAGCTCACCAT
Human-β-actin-R	TCGTCGCCCACATAGGAATC
